# Global transcriptomic analysis reveals candidate genes associated with different phosphorus acquisition strategies among soybean varieties

**DOI:** 10.3389/fpls.2022.1080014

**Published:** 2022-12-19

**Authors:** Tongli Yang, Songhua Yang, Zhu Chen, Yuechen Tan, Roland Bol, Honglang Duan, Jin He

**Affiliations:** ^1^ College of Agriculture, Guizhou University, Guiyang, China; ^2^ Agricultural Ecological Environment and Resources Protection Station of Bijie Agricultural and Rural Bureau, Guiyang, China; ^3^ Institute of Ecological Conservation and Restoration, Chinese Academy of Forestry, Beijing, China; ^4^ Institute of Bio- and Geosciences, Agrosphere (IBG-3), Forschungszentrum Jülich GmbH, Jülich, Germany; ^5^ School of Natural Sciences, Environment Centre Wales, Bangor University, Bangor, United Kingdom; ^6^ Institute for Forest Resources & Environment of Guizhou, College of Forestry, Guizhou University, Guiyang, China

**Keywords:** soybean, root phosphorus acquisition strategy, root diameter, acetyl-CoA, transcriptome

## Abstract

**Introduction:**

Soybean adapts to phosphorus-deficient soils through three important phosphorus acquisition strategies, namely altered root conformation, exudation of carboxylic acids, and symbiosis with clumping mycorrhizal fungi. However, the trade-offs and regulatory mechanisms of these three phosphorus acquisition strategies in soybean have not been researched.

**Methods:**

In this study, we investigated the responses of ten different soybean varieties to low soil phosphorus availability by determining biomass, phosphorus accumulation, root morphology, exudation, and mycorrhizal colonization rate. Furthermore, the molecular regulatory mechanisms underlying root phosphorus acquisition strategies were examined among varieties with different low-phosphorus tolerance using transcriptome sequencing and weighted gene co-expression network analysis.

**Results and discussion:**

The results showed that two types of phosphorus acquisition strategies—“outsourcing” and “do-it-yourself”—were employed by soybean varieties under low phosphorus availability. The “do-it-yourself” varieties, represented by QD11, Zh30, and Sd, obtained sufficient phosphorus by increasing their root surface area and secreting carboxylic acids. In contrast, the “outsourcing” varieties, represented by Zh301, Zh13, and Hc6, used increased symbiosis with mycorrhizae to obtain phosphorus owing to their large root diameters. Transcriptome analysis showed that the direction of acetyl-CoA metabolism could be the dividing line between the two strategies of soybean selection. ERF1 and WRKY1 may be involved in the regulation of phosphorus acquisition strategies for soybeans grown under low P environments. These findings will enhance our understanding of phosphorus acquisition strategies in soybeans. In addition, they will facilitate the development of breeding strategies that are more flexible to accommodate a variety of production scenarios in agriculture under low phosphorus environments.

## Introduction

Phosphorus deficiency is the main nutritional factor limiting crop production. The fertilizer efficiency of phosphate is much lower because it is easily precipitated by aluminum, iron, and calcium in the soil (Péret et al., 2011). Excessive phosphorus application causes agricultural and environmental pollution and increases the consumption of non-renewable phosphate rock resources (López-Arredondo et al., 2014). There is increasing recognition that improved crop adaptation to low soil fertility is a cornerstone of the second agricultural revolution (White et al., 2013). Tapping the soil phosphorus acquisition potential of the root and selecting and breeding phosphorus-efficient varieties is an economical and sustainable way for agricultural development.

Classical root resource economics is based on the “leaf economics spectrum” (Wright et al., 2004; Shipley et al., 2006). These studies suggest that plants with a “rapid resource acquisition” strategy have longer, thinner roots, which help them in acquiring more resources with less biomass investment. In contrast, plants with a “conservation of resources” strategy have thicker and denser root systems to achieve longer lifespans and longer returns on investment (Freschet et al., 2010; Reich, 2014). However, root systems have a more complex strategy for obtaining subterranean resources than do leaves (Kramer-Walter et al., 2016; Ma et al., 2018). After analyzing a global dataset of root traits for 1810 species, a new collaborative gradient, which consists of specific root length (SRL), mycorrhizal colonization rate, and root diameter, was discovered (Bergmann et al., 2020). The collaborative gradient and the classical “conservation” gradient together constitute a new economic space for the root system. In addition, root exudation is also a phosphorus acquisition strategy to be considered in farming (Chen and Liao, 2016; Wen et al., 2022). Among these root exudations, organic acids and purple acid phosphatase activate insoluble inorganic and organic phosphorus in the root interface, thereby increasing phosphorus effectiveness (Hinsinger et al., 2003; Preece and Peñuelas, 2020; Wang and Lambers, 2020). In addition, flavonoids and glutanolactones are signaling molecules that establish symbiosis between roots and mycorrhizae (Lopez-Raez et al., 2017; Sasse et al., 2018). Overall, upon low phosphorus stress, plants generate three root phosphorus acquisition strategies, viz. remodeling of root morphology, symbiosis with mycorrhizal fungi, and increasing root exudation (Ham et al., 2018; Wen et al., 2022). However, the specific strategy or strategies that the photosynthetic assimilation products are allocated to may imply a reduction in carbon allocation from the other strategies, that is, a weakening of the other strategies (Raven et al., 2018; Lu et al., 2022). Currently, most studies focus on independent changes or paired combinations of plant exudation, root morphology, and mycorrhizal symbiosis (Lyu et al., 2016; Deng et al., 2017; Wang et al., 2020). By simultaneously detecting three root function traits, we will develop more effective phosphorus acquisition strategies that help crops to develop more effective phosphorus acquisition strategies.

Over the past few decades, a set of genes that improve root phosphorus acquisition has been functionally characterized, indicating the complexity of plant Pi signaling networks (Ham et al., 2018; Dissanayaka et al., 2021). Numerous studies have elucidated the mechanism by which SPX (SYG1/Pho81/XPR1) protein and Phosphate starvation response regulator (PHR) complex (SPX-PHR) coordinates the phosphorus efficient transporter (*PHT*) in response to low phosphorus stress through IPS1-miRNA399-PHO2 (Wege et al., 2016; Zhang et al., 2016). Recent studies have shown that SPX-PHR can also regulate Reduced Arbuscular Mycorrhiza 1 (*OsRAM1*) and WRINKLED5a (*OsWRI5A*), which are key transcription factors that mediate fatty acid synthesis and transport as well as the expression of a phosphate transporter (*OsPT11*) and ammonium transporter (*OsAMT*), which are essential for nutrient exchange at the peri-arbuscular interface (Shi et al., 2021; Wang et al., 2022). Balzergue et al. (2017) demonstrated that under Pi starvation, plants acquire phosphorus through Sensitive to Proton Rhizotoxicity and Aluminum-activated Malate Transporter 1 (STOP1-ALMT1) and Phosphate deficiency response 2 and low phosphate response 1 (PDR2-LPR1) signaling pathways to promote malate exudation to the root, which hinders cell proliferation in root tip meristematic tissues and further alters root architecture (Mora-Macías et al., 2017; Balzergue et al., 2017). This evidence implies that the three phosphorus acquisition strategies are not regulated individually but in the form of shared core regulatory factors as well as coordinated multiple feedback mechanisms (Branscheid et al., 2010; Castrillo et al., 2017); nevertheless, there are several knowledge gaps regarding the regulatory mechanisms underlying root phosphorus acquisition strategies.

As an important source of protein and oil, soybeans (*Glycine max*) are grown throughout the world (Herridge et al., 2008). Low phosphorus effectiveness in soils is the most important limiting factor for soybean production (Mo et al., 2019). Soybean adapts to phosphorus-deficient soils through three important phosphorus acquisition strategies (Guo et al., 2008; Wang et al., 2016; Peng et al., 2018). However, the trade-offs and regulatory mechanisms of these three phosphorus acquisition strategies remain to be explored in soybean. Based on previous studies (Zhang et al., 2021; Yang et al., 2022), we selected 10 soybean varieties with differences in phosphorus efficiency as experimental plants Then the biomass, phosphorus accumulation, root morphology, root exudation, and mycorrhizal infestation rate were measured in 10 soybean varieties under low phosphorus stress conditions. Subsequently, the low phosphorus tolerant varieties Qd11 and Zh13 and the low phosphorus sensitive variety Nm were further selected for transcriptome sequencing. The objectives of this study were to investigate whether phosphorus acquisition strategies differ among the 10 soybean varieties and how these phosphorus acquisition strategies respond to soil phosphorus availability; furthermore, we sought to fill in the gaps in our understanding of how phosphorus acquisition strategies are linked in soybean through transcriptome analysis.

## Materials and methods

### Plant materials and growth conditions

According to previous studies (Zhang et al., 2021; Yang et al., 2022), the following 10 soybeans (*Glycine max*) varieties with gradients in phosphorus acquisition efficiency were used in this study: Qian Dou 11 (Qd11), Shang Dou 1013 (Sd), Niu Mao Soybean (Nm), Zhonghuang 13 (Zh13), Qixing 1 (Qx1), Hua Chun 6 (Hc6), Zhonghuang 30 (Zh30), Hua Xia 3 (Hx3), Ai Xuan (Ax), Hua Xia 2 (Hx2), Suiyang bean (Sy), Zhonghuang 301 (Zh301), and Zhonghuang 61 (Zh61).

The experiment included two phosphorus treatments—normal phosphorus (NP) and low phosphorus (LP). The experiment began on April 1, 2021, in the greenhouse at Guizhou University, Guiyang City, Guizhou Province, China. A plastic bucket with a diameter of 26 cm and a height of 35 cm was used as a potting bowl and filled with 15 kg of mixed soil (9:1, riverside sand: low phosphorus availability soil). The properties of mixed soil were as follows: pH 8.9 (soil: water, 1:2.5), 2.4 mg·kg^-1^ Olsen-P, 3.1 g·kg^-1^ organic carbon, 2.8 mg·kg^-1^ alkaline soluble nitrogen, and 100 mg·kg^-1^ exchangeable K. Every pot was seeded with six to ten full-grained, uniformly sized seeds. The number of seedlings was set to four plants per pot after the first compound leaves appeared. Different treatments were initiated when seedlings reached two leaves and one heart, and the treatment was conducted by adding a nutrient solution. The nutrient composition of the NP treatment was 0.5 mmol·L^-1^ KH_2_PO_4_, 1.5 mmol·L^-1^ KNO_3_, 1.2 mmol·L^-1^ Ca(NO_3_)_2_, 0.4 mmol·L^-1^ NH_4_NO_3_, 0.3 mmol·L^-1^ K_2_SO_4_, 0.5 mmol·L^-1^ MgSO_4_, 25.0 μmol·L^-1^ MgCl_2_, 0.3 mmol·L^-1^ (NH_4_)_2_SO_4_, 0.04 mmol·L^-1^ EDTA-Fe, 1.5 μmol·L^-1^ MnSO_4_, 0.5 μmol·L^-1^ CuSO_4_, 1.5 μmol·L^-1^ ZnSO_4_, 2.5 μmol·L^-1^ Na_3_BO_3_, 0.15 μmol·L^-1^ (NH_4_)_2_MoO_4_, and 0.1 μmol·L^-1^ CoCl_2_, pH was adjusted to 5.9–6.5. The concentration of KH_2_PO_4_ was 0.02 mmol·L^-1^ in the LP treatment, whereas other components remained unchanged, and the missing K^+^ was supplemented with KCl. The nutrient solution was watered thoroughly (1 L·pot^-1^) every 3 days over a 16-day treatment. Each treatment was performed in three replicates, and a completely random design was used to arrange the pots. Routine management ensured that other experimental conditions were consistent.

### Determination of carboxylate and acid phosphatase exudation

At harvest, the entire root system was carefully removed from pots, and the soil was gently shaken off the roots. Then, the roots were transferred to a beaker that contained 50 ml of 0.5 mmol·L^-1^ CaCl_2_. Two drops of thymol (20 g·L^-1^) were added to the CaCl_2_ solution to inhibit the growth of microorganisms. The roots were soaked repeatedly in the solution, taking care to minimize damage to the roots. According to Shen et al., (2003) the collected solution was filtered using a 0.22 m filter membrane, and the carboxylate content was assessed using high-performance liquid chromatography (1260 Infinity, Agilent, Santa Clara, CA, USA). Using the method outlined by Liu et al., (2004) the activity of root-secreted acid phosphatase (APase) was assessed.

### Determination of morphological characteristics of roots

After finishing the sampling of root exudations, roots were carefully washed. Images of root morphology were obtained using a scanner (Perfection V850 Pro-type, Epson, Long Beach, CA, USA). To obtain the morphological characteristics of roots, the images were processed with the root analysis system (Win-RHIZO, Regent Instructions, Quebec, Canada).

### Determination of mycorrhizal infestation rate

The cleaned roots were cut into 1 cm length root segment and placed in the Formalin-Aceto-Alcohol fixative for low-temperature storage. To determine the mycorrhizal infestation rate, the roots were first digested with 10% KOH at 90°C for 60 min, cooled, and poured out of the KOH solution. Then, the roots were cleaned with distilled water 2–3 times and then acidified with 2% HCl for at least 30 min, following which they were cleaned with distilled water 3–5 times. The roots were then stained with 0.05% acidic magenta staining solution in a water bath at 90 °C for 20 min to 1 h, after which the staining solution was drained, and the roots were decolorized by adding lactic acid glycerol solution (Phillips and Hayman, 1970). Finally, 30 root segments were randomly selected and placed in 30% glycerol soaked in slides, covered with coverslips, observed under a microscope (BX51, Olympus, Tokyo, Japan), and counted using the grid cross method. The mycorrhizal infestation rate was calculated according to the method of Trouvelot et al. (1986).

### Determination of plant biomass and phosphorus accumulation

The plants were cleaned and dried at 105°C for 30 min, and then the temperature was adjusted to 75°C to dry to a constant weight. The dry weight was then measured in an analytical balance. The samples of dried plants were crushed and sieved through a 0.25 mm sieve; then, the samples were decocted using the H_2_SO_4_-H_2_O_2_ method. Phosphorus content was then determined using the molybdenum blue method (Xue et al., 2018). The shoot and root biomass were multiplied by their phosphorus content separately, and the phosphorus accumulation of the shoot and root was summed to obtain the phosphorus accumulation of the whole plant.

### Transcriptome analysis of root samples

After 16 d of NP or LP treatment, the roots of three plants with the same growth conditions were taken, washed, and blotted dry. The roots were frozen in liquid nitrogen and then stored in a −80°C refrigerator until transcriptome analysis. Total root RNA was isolated using TRIzol reagent (Invitrogen, Carlsbad, CA, USA). For the construction of libraries, approximately 1 μg of RNA per sample was used. The TruSeq RNA sample prep kit (Illumina, San Diego, CA, USA) was used to prepare the libraries. Sequencing of the cDNA libraries was conducted using an Ilumina NovaSeq 6000 sequencer (Illumina). Using fastp, raw sequences were filtered and quality-controlled (Chen et al., 2018). HISAT2 (Kim et al., 2015) was used to splice the clean reads to the soybean genome (Williams82 a2.v1) for comparison. StringTie was used to assign each sequence to individual samples (Pertea et al., 2015). The RNA-Seq by Expectation-Maximization (RSEM) software was used to calculate transcripts per million (TPM) reads for each sample (Li and Dewey, 2011). The Edger software was used to identify the differentially expressed genes (DEGs) by | log2 (Fold change) | ≥2 and corrected P-adjust < 0.05 (Robinson et al., 2010). Gene ontology (GO) was performed using Goatools (Conesa et al., 2005). Kyoto Encyclopedia of Genes and Genomes (KEGG) pathway analyses were performed using KOBAS (Xie et al., 2011). Transcriptome raw data have been deposited in the Sequence Read Archive of the National Center for Biotechnology Information (NCBI) database (bioproject accession number: PRJNA867332).

### Statistical analysis

The effects of soybean varieties, P treatment, and their interaction on the plant growth parameters and root phosphorus acquisition traits were assessed *via* a two-way ANOVA using Data Processing System (version 7.05, Zhejiang University, Hangzhou, China). Using DPS, Student’s *t*-tests were performed to determine whether significant differences occurred among the treatments. Principal component analysis (PCA) was conducted using the Vegan (Oksanen et al., 2015), and PCA plots were produced using the ggplot2 (Wickham, 2016). WGCNA R software package (version 4.0.2, Langfelder and Horvath, 2008) was used to construct co-expression networks. Parameters were set to networkType = signed, power = 9, minModuleSize = 30. Heatmaps were created using the TBtools software (Chen et al., 2020). An overview of the analysis pipelines implemented in this study is presented in **Figure S1**.

## Results

### Responses of root phosphorus acquisition strategies to soil phosphorus availability

**Figure 1:**
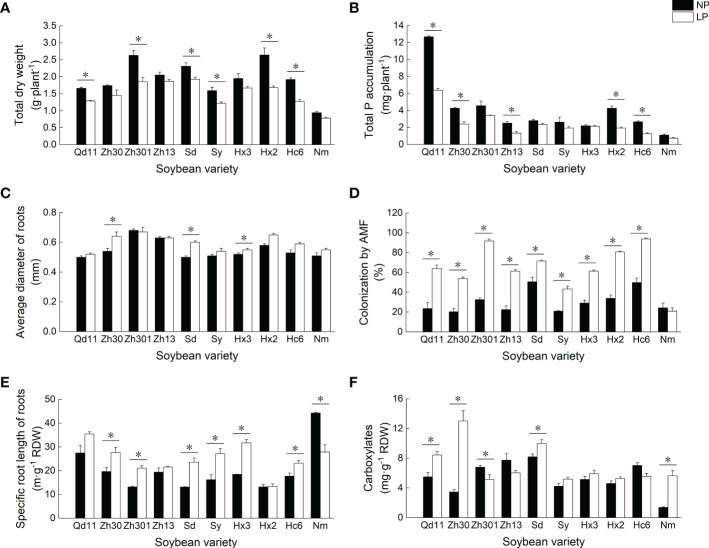
Effects of Pi availability among 10 varieties on the soybean physiology. Effects of Pi availability among 10 varieties on the **(A)** total dry weight; **(B)** total P accumulation; **(C)** average diameter of roots; **(D)** colonization by arbuscular mycorrhizal fungi; **(E)** specific root length of roots; and **(F)** amounts of carboxylates in the rhizosheath. Varieties abbreviations: Qd11, Qian Dou 11; Zh13, Zhonghuang 13; Nm, Niu Mao Soybean; Sd, Shang Dou 1013; Qx1, Qixing 1; Hc6, Hua Chun 6; Zh30, Zhonghuang 30; Hx3, Hua Xia 3; Ax, Ai Xuan; Hx2, Hua Xia 2; Sy, Suiyang bean; Zh301, Zhonghuang 301; and Zh61, Zhonghuang 61. LP, low soil phosphorus availability; NP, normal soil phosphorus availability. *Denotes significant difference between P treatments (based on LSD, *p* ≤ 0.05).

Low soil phosphorus availability resulted in the reduction of soybean biomass and phosphorus accumulation (**Figures 1A, B**). As compared to NP, LP treatment reduced the biomass and phosphorus accumulation of 10 soybean varieties by 4–37% and 3–55%, respectively. Among all varieties, Qd11 showed the highest phosphorus accumulation of 6.4 mg per plant under LP treatment, whereas Nm showed the lowest phosphorus accumulation of 0.7 mg per plant.

In response to low phosphorus availability, most varieties showed consistent trends in root traits— SRL (**Figure 1E**), root surface area (**Table S1**), arbuscular mycorrhizal fungi (AMF) colonization rate (**Figure 1D**), and APase (**Table S1**) increased, whereas root tissue density decreased (**Table S2**). The AMF colonization rate of roots after LP treatment increased by 41–183% compared to those with NP treatment. With LP treatment, varieties with thicker roots, such as Zh301 and Hx2, tended to have higher AMF colonization rates (89–92%) than those of the varieties with thinner root systems (**Figure 1D**). In contrast, varieties with thinner roots (e.g., Qd11, Sy, and Nm) had low AMF colonization rates of 21–64%. Root exudation of carboxylates was increased in most varieties with LP treatment, which was particularly prominent in varieties with higher SRL (e.g., Qd11 and Zh30; **Figure 1F**). The total amounts of carboxylates secreted by the roots of Qd11 and Zh30 were 54% and 277%, respectively, higher with LP treatment than with NP treatment owing to the elevated exudation of malate, citrate, oxalate, and lactate (**Table S2**). In contrast, root exudation of carboxylates from varieties with lower SRL (e.g., Zh301 and Zh13) was reduced with LP treatment (**Figure 1F**).

**Figure 2:**
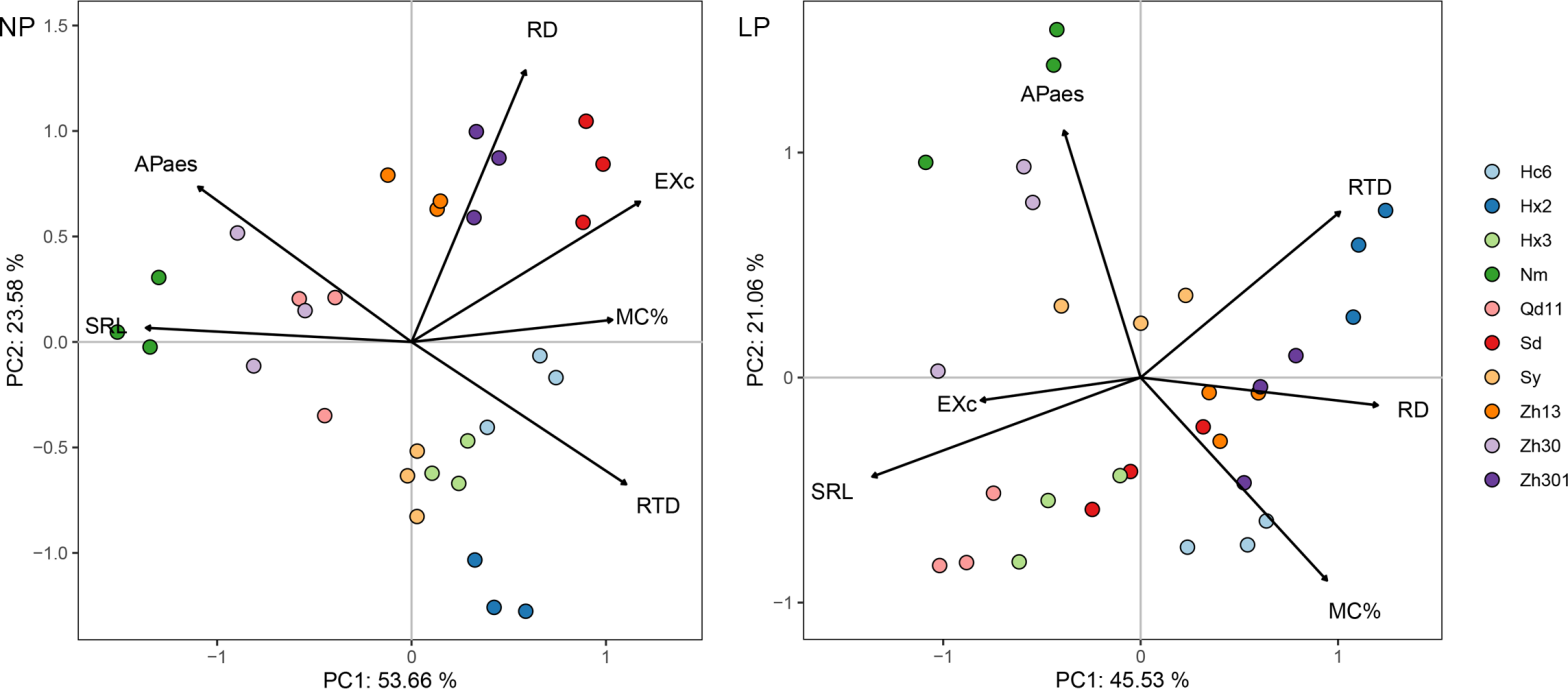
Principal component analysis of root functional traits for 10 varieties in response to normal (NP) and low (LP) soil phosphorus levels. Root trait RD, average diameter of roots; SRL, specific root length; RTD, root tissue density; MC%, colonization by arbuscular mycorrhizal fungi; EXc, amounts of carboxylates in the rhizosheath; APase, acid phosphatase activity in the rhizosheath. Varieties abbreviation: Qd11, Qian Dou 11; Zh13, Zhonghuang 13; Nm, Niu Mao Soybean; Sd, Shang Dou 1013; Qx1, Qixing 1; Hc6, Hua Chun 6; Zh30, Zhonghuang 30; Hx3, Hua Xia 3; Ax, Ai Xuan; Hx2, Hua Xia 2; Sy, Suiyang bean; Zh301, Zhonghuang 301; and Zh61, Zhonghuang 61.

PCA of the six root functional characteristics of the 10 soybean varieties with LP treatment showed that the first component (PC1) accounted for 46% of the variation and was primarily determined by two root morphological characteristics (SRL and root diameter) and root carboxylic acid exudation; the second component (PC2) accounted for 21% of the variation and was dominated by APase and colonization by AMF (**Figure 2**). Most varieties with thicker roots (e.g., Hx2, Zh301, Zh13, and Hc6) clustered and scattered in the directions of RTD, RD, and colonization by AMF. The varieties with thinner roots (e.g., Qd11 and Hx3) were in the root carboxylic acid exudates direction. With NP treatment, 78% of the total variation could be explained by the first two components of PCA (**Figure 1B**). Most varieties with thicker root varieties were distributed on the positive axis of PC1, which was dominated by colonization by AMF and root carboxylic acid exudation, whereas the negative axis was dominated by Nm, Zh30, and Qd11.

### Transcriptome analysis of soybean root response to phosphorus availability

**Figure 3:**
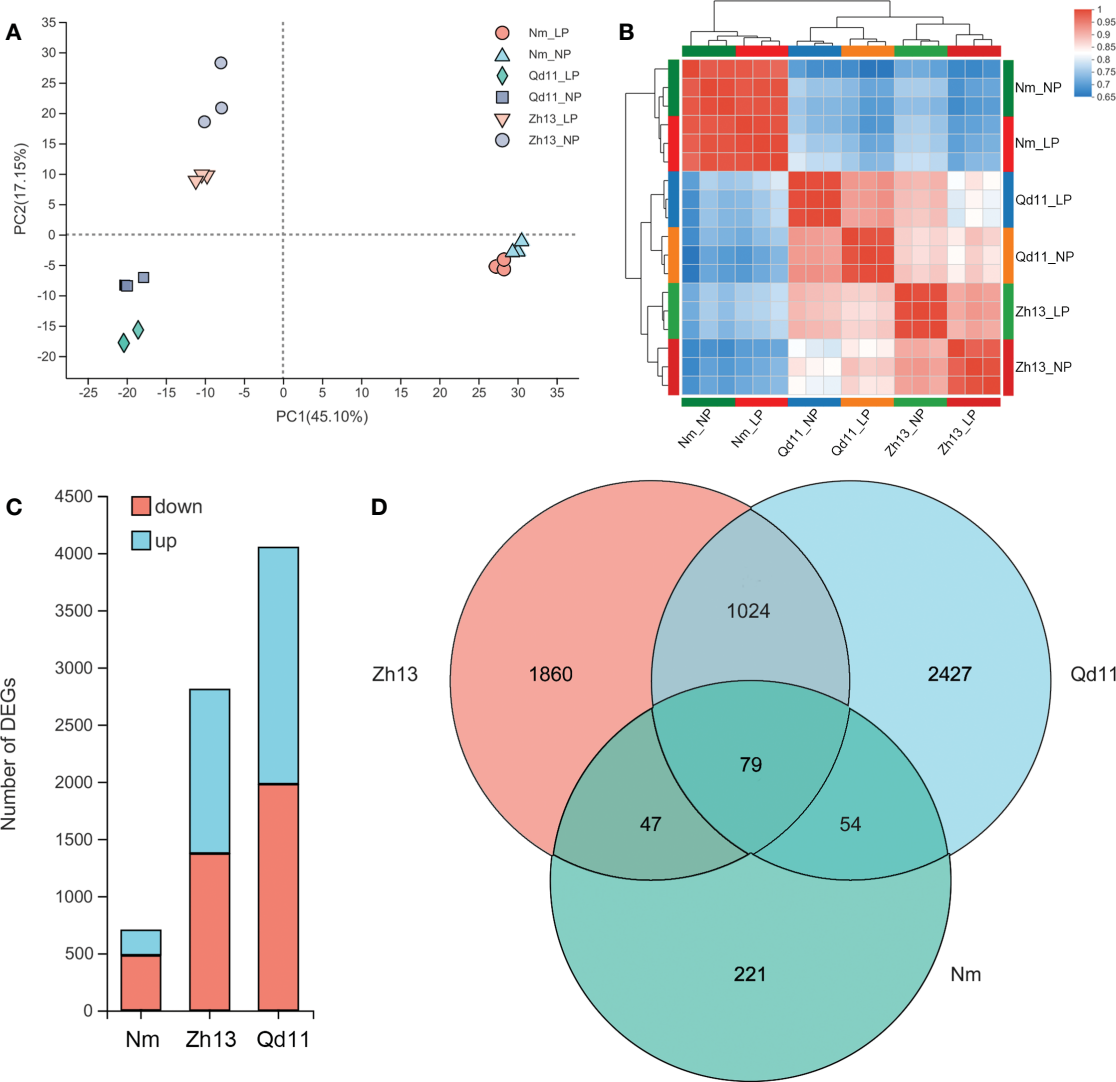
Principal component analysis of the genes expressed in Qd, Zh13, and Nm in response to normal (NP) and low (LP) soil phosphorus availability **(A)**; Pearson correlations for Qd, Zh13, and Nm in response to NP and LP treatments. The colors represent the distances among the samples **(B)**; principal component analysis of the expressed genes **(B)**; number of differentially expressed genes in each variety **(C)**; Venn diagram of the three varieties of differentially expressed genes **(D)**. Varieties abbreviations: Qd, Qian Dou 11; Zh13, Zhonghuang 13; Nm, Niu Mao Soybean.

To investigate the mechanisms regulating the strategies adopted by soybean roots for phosphorus acquisition under low soil phosphorus availability, we selected three varieties, Qd11, Zh13, and Nm, with differential root phosphorus acquisition efficiency and strategies for transcriptomic analysis (**Figures 1**, **2**). Transcriptomic analysis of 18 soybean root samples yielded a total of approximately 959 million clean reads, and approximately 856 million clean reads were mapped to the soybean reference genes (**Table S3**). PCA analysis showed that the transcriptomes of Qd11 and Zh13 were located on the negative axis of the PC1 under both LP and NP treatments, whereas those of Nm were all located on the positive axis of the PC1 (**Figure 3A**), suggesting that the gene expression profile of Nm was markedly different from that of the other two varieties. Moreover, the Pearson correlation coefficients of all samples in Qd11 and Zh13 were 0.92–0.94 and 0.90–0.93, respectively, whereas those of Nm were 0.97–0.99, which were much higher than those of Qd11 and Zh13 (**Figure 3B**). This indicated that the differences in LP and NP gene expression profiles were smaller in Nm than in Qd11 and Zh13.

**Figure 4:**
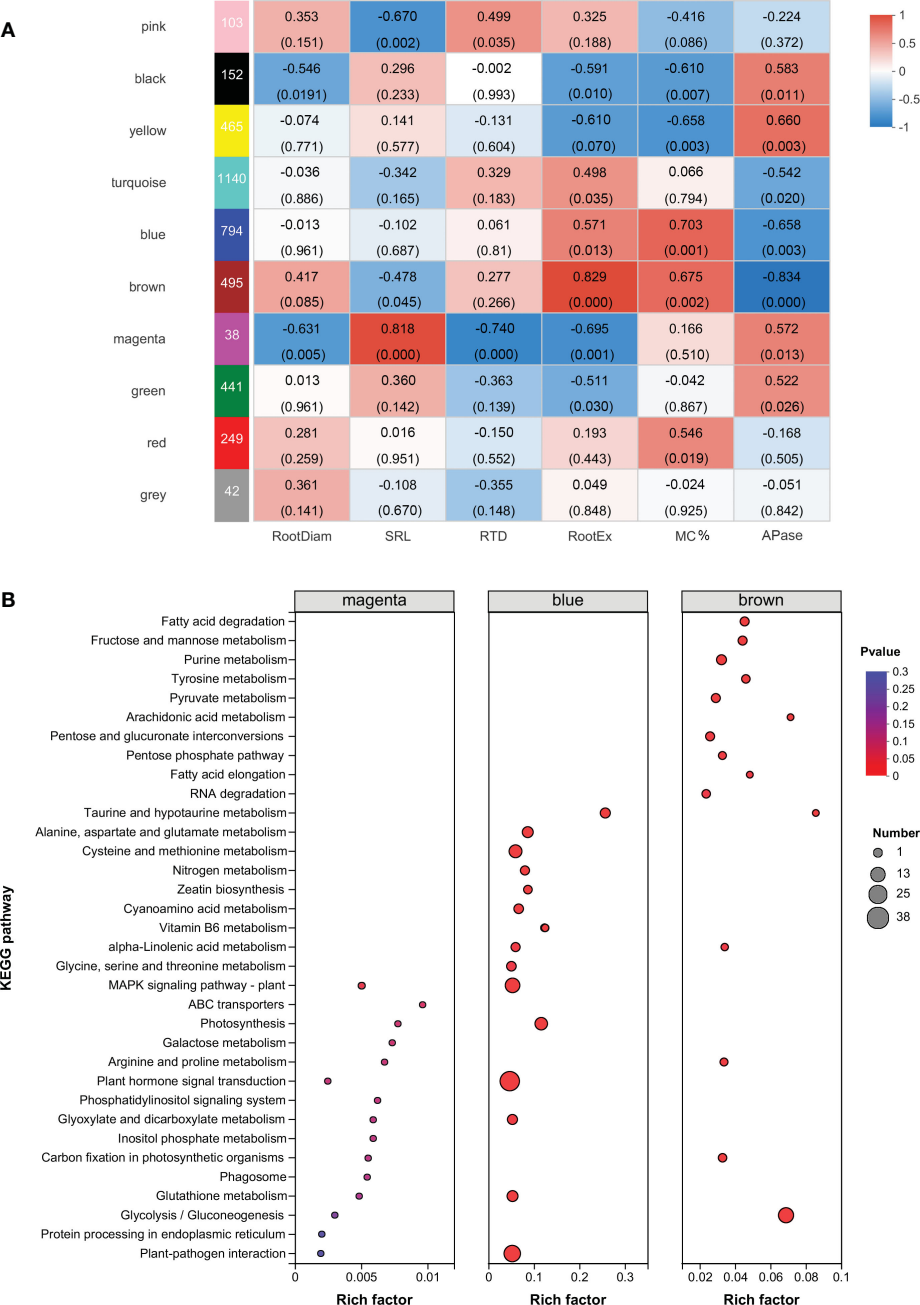
Identification of modules involved in the acquisition of root phosphorus. **(A)** Weighted Gene co-Expression Network Analysis (WGCNA) of deferentially expressed genes. On the left side, ten modules are displayed. p-values are shown in parentheses. The color scale on the right side shows module-trait correlation from −1 (blue) to 1 (red). **(B)** KEGG enrichment analysis of sample-specific modules. The color and size of the circle indicate the significance level (P value) and the number of target genes involved, respectively. Root trait abbreviations: RootDiam, average diameter of roots; SRL, specific root length; RTD, root tissue density; RootEX, the amount of carboxylates in the rhizosheath; MC%, colonization by arbuscular mycorrhizal fungi; APase, acid phosphatase activity in the rhizosheath.

DESeq2 analysis showed that the Qd11 root system had the most DEGs at 4049, of which 2068 genes were upregulated, and 1981 were downregulated (**Figure 3C**, **Figure S2**). Zh13 had 2808 DEGs, with 1434 genes upregulated and 1374 genes downregulated. Nm had the lowest number of DEGs, with only 701 DEGs detected, which was only 17% and 25% of the number of DEGs in Qd11 and Zh13. Seventy-nine DEGs were detected jointly in all three varieties (**Figure 3D**); these genes are the most important response genes in soybean in the face of low soil phosphorus availability. A total of 1024 DEGs were detected in both Qd11 and Zh13 (**Figure 3D**); these genes are potentially phosphorus-efficient genes in soybean. KEGG analysis revealed the top 20 significantly enriched key biological pathways in the three soybean varieties (**Figure S3**), with the majority of DEGs enriched in glycolysis/gluconeogenesis, phenylpropanoid biosynthesis, MAPK signaling pathway, and plant-pathogen interaction (**Figure 4**).

Transcription factors are critical for the regulation of downstream genes under different soil phosphorus availability. Among the 5712 DEGs, 510 were annotated as transcription factors (TFs), including 85 ERF family members, 56 MYB family members, bHLH family members, 40 NAC family members, and 37 WRKY family members (**Table S4**). The highest number of TFs was detected in the DEGs of Qd11, with 361, and the least number was detected in the DEGs of Nm, which had only 19 (**Table S4**). Meanwhile, several gene families are involved in Pi acquisition and homeostasis, including seven phosphate transporters, three purple acid phosphatases (PAP), six aluminum-activated malate transporters (ALMT), one aluminum-activated citrate transporters (MATE), 26 ABC transporters, four PHR, one SPX, and one microRNA MIR399b (**Table S5**), were also identified as responsive to Pi starvation.

### Weighted gene co-expression network analysis of soybean root phosphorus acquisition strategies

To explore the gene regulatory modules and key genes associated with root phosphorus acquisition strategies, we analyzed 5712 DEGs using the WGCNA analysis. A correlation analysis of modules and root morphological traits showed that the magenta module (*r* = 0.82, *p* = 0.00003) had the strongest correlation with SRL (**Figure 4A**). The central transcription factors ethylene receptor (ETR, involved in ethylene signaling), inositol-tetrakisphosphate (ITPK1; involved in phosphatidylinositol signaling response), serine/threonine protein phosphatase (PP2C33), and other transcription factors (MYB family transcription factor EFM, WRKY1, dehydration-responsive element binding protein 3) were identified in the magenta module. These proteins may be involved in the regulation of upstream signaling of soybean roots in response to low soil phosphorus availability. In addition, glutathione S-transferase (GST), disease resistance-related calreticulin (CRT), pathogenesis-related protein 1 (PR1), ATP-binding cassette subfamily B (MDR/TAP), and aldose 1-epimerase (renamed galactose mutarotase, GALM) were identified in the magenta module.

The blue module showed the strongest correlation with colonization by AMF (*r* = 0.70, *p* = 0.0001, **Figure 4A**). KEGG enrichment analysis indicated that the blue module significantly enriched plant hormone signal transduction, plant-pathogen interaction, and the MAPK signaling pathway. The brown module was correlated with both colonization by AMF (*r* = 0.675, *p* = 0.002) and root carboxylate exudation (*r* = 0.509, *p* = 0.031). The glycolysis/gluconeogenesis pathway was significantly enriched in the brown module (**Figure 4B**; **Table S6**). In addition, inositol polyphosphate 5-phosphatase (INPP5B/F) was detected in the blue module, and inositol-phosphate phosphatase (VTC4) and phosphatidylinositol phospholipase C (PLC) were detected in the brown module. The strongest correlation was observed between root acid phosphatase exudation and the yellow module (*r* = 0.66, *p* = 0.003).

## Discussion

We aimed to investigate whether phosphorus acquisition strategies differ among 10 soybean varieties and how these phosphorus acquisition strategies respond to soil phosphorus availability; furthermore, we sought to fill in the gaps in our understanding of how phosphorus acquisition strategies collaborate in soybean through transcriptome analysis. Our results suggested considerable intraspecific variation in phosphorus acquisition strategies amongst the 10 soybean varieties. The 10 soybean varieties were divided into two types, which adopted two opposing phosphorus acquisition strategies under low phosphorus availability—”outsourcing” and “do-it-yourself” (Bergmann et al., 2020; Lambers, 2022). The “do-it-yourself” strategy was adopted by the soybean varieties represented by Qd11, Zh30, and Sd. Under low phosphorus availability, SRL and carboxylic acid exudation were substantially higher in the “do-it-yourself” varieties than in the “outsourcing” varieties. This increased the contact area of the root system with the soil and improved the mobility of insoluble phosphate around the root system, thus promoting the acquisition of phosphorus. However, the analysis of Zh301, Zh13, and Hc6 varieties revealed that their ability to exude carboxylic acids was reduced rather than increased under low phosphorus availability. Instead, these soybean varieties had higher colonization by AMF than the varieties adopting the “do-it-yourself” strategy. We define varieties that adopt this strategy as outsourcing varieties. Outsourcing varieties had a larger average root diameter than do-it-yourself varieties, which made it difficult for them to adjust their root architecture to obtain sufficient phosphorus in response to low phosphorus. However, the larger average root diameter provides an adequate in-root habitat for AMF (Valverde-Barrantes et al., 2016). AMF very slender mycelium, and therefore symbiosis with AMF enhances the spatial effectiveness of phosphorus in the soil for the roots. Therefore, increasing the mycorrhizal infestation rate becomes the preferred strategy for thicker root varieties. PCA confirmed the positive correlation between mycorrhizal infestation rate and root diameter, and WGCNA further confirmed the synergistic regulatory mechanism between root architecture and the mycorrhizal infestation process. Overall, for these 10 soybean varieties, varieties with thinner roots exhibited higher SRL and exuded greater amounts of carboxylates in response to LP. Conversely, varieties with thicker roots had a greater AMF colonization rate.

When several species were studied together, SRL was found to be negatively correlated with root-secreted organic acid content (Wen et al., 2019; Honvault et al., 2021). Studies on Arabidopsis have shown that under low phosphorus, the transcription factor STOP1 activates the expression of ALMT1 (Mora-Macías et al., 2017). ALMT1 secretes malic acid extracellularly and induces callus formation within the main root stem cell niche (SCN), which ultimately obstructs root elongation (Balzergue et al., 2017). However, to date, the inhibition of root elongation by malic acid has only been detected in plants with thinner roots, such as *Arabidopsis* and *Sorghum*. We found that in soybean, SRL was positively correlated with the content of root-secreted carboxylic acid. This is consistent with the findings of studies on thicker-rooted plants such as soybean (Zhou et al., 2016), chickpea (Wen et al., 2020), and temperate trees (Wang et al., 2021; Akatsuki and Makita, 2020). The mechanisms regulating root morphology and carboxylic acid exudation may differ among species. *Arabidopsis* and grasses may have evolved in environments with relatively high soil phosphorus effectiveness, and they belong to non-phosphorus-mobilizing species with negative regulatory mechanisms between carboxylic acid exudation and root elongation (Lambers et al., 2008). Plants growing in extremely phosphorus-deficient soils, such as soybean, faba bean, alfalfa, and temperate trees, are phosphorus-mobilizing species (Lambers et al., 2018; Akatsuki and Makita, 2020) that can increase both SRL and carboxylic acid exudations under low soil phosphorus availability (Yu et al., 2020), resulting in greater tolerance to low phosphorus (Hayes et al., 2021).

**Figure 5:**
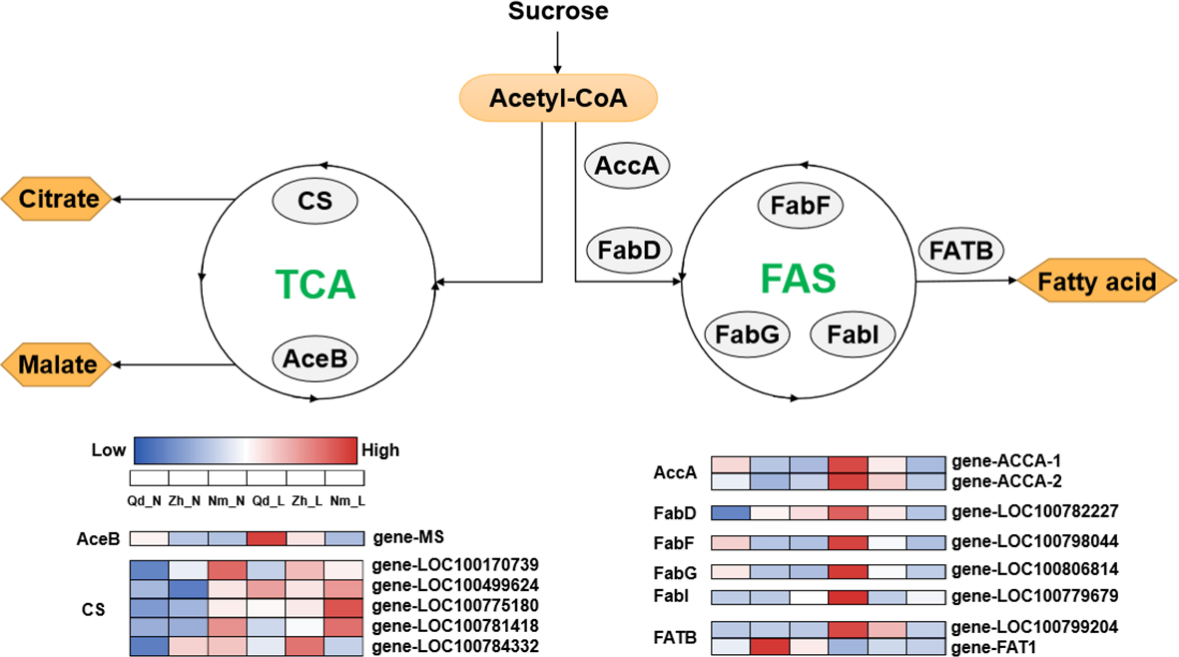
Deferentially expressed genes associated with the fatty acid biosynthesis and citrate cycle pathways. accA, acetyl-CoA carboxylase carboxyl transferase; FabD, [acyl-carrier-protein] S-malonyltransferase; FabF, 3-oxoacyl-[acyl-carrier-protein] synthase II; FabG, 3-oxoacyl-[acyl-carrier protein] reductase; FabI, enoyl-[acyl-carrier protein] reductase I; FATB, fatty acyl-ACP thioesterase B; AceB, malate synthase; CS, citrate synthase. The heat map shows the expression patterns of differentially expressed genes related to the fatty acid biosynthesis and citrate cycle pathways in Qd11, Zh13, and NM at different P treatments. Enzyme names are shown in the gray circle. The color bar from blue to red indicates upregulation; the color bar from red to blue indicates downregulation. Varieties abbreviations: Qd, Qian Dou 11; Zh13, Zhonghuang 13; Nm, Niu Mao Soybean; L, low soil phosphorus availability; N, normal soil phosphorus availability.

**Figure 6:**
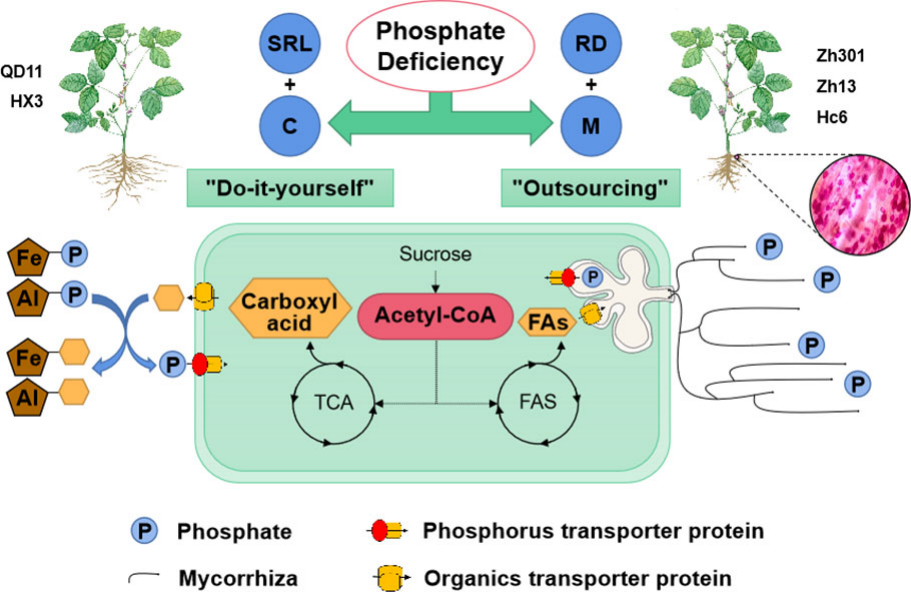
Schematic representation of phosphorus acquisition strategies in soybean under phosphate deficiency. Under low phosphorus availability, two types of phosphorus acquisition strategies—”do-it-yourself” and “outsourcing” were used by soybean varieties. The “do-it-yourself” varieties obtained sufficient phosphorus by increasing their SRL and secreting carboxylic acids (SRL+C). By secreting carboxylic acids, roots dissolve phosphorus immobilized by iron and aluminum, which elevates the concentration of phosphorus in soil water. Additionally, the “do-it-yourself” varieties had larger SRLs, indicating a larger root surface area, which further promoted phosphorus absorption. Owing to their larger root diameters, the “outsourcing” varieties utilized increased symbiosis with mycorrhizal fungi to obtain phosphorus (RD + M). The direction of acetyl-CoA metabolism might be the dividing line between the “outsourcing” and “do-it-yourself” strategies of soybean. The “do-it-yourself” varieties converted acetyl-CoA into carboxylic acid, primarily through the TCA cycle, whereas the “outsourcing” variety converted it into fatty acids, which were used to exchange phosphorus with mycorrhizal fungi. SRL, specific root length; RD, average diameter of roots; M, Mycorrhizal fungi; C, Carboxyl acid; FAs, Fatty acids; TCA, Tricarboxylic Acid Cycle; FAS, Fatty Acid Synthase.

The PCA showed a negative correlation between colonization by AMF and carboxylic acid exudation. Both carboxylic acid exudation and mycorrhizal colonization require large amounts of substantial carbohydrates; therefore, the metabolism of carbohydrates determines the relationship between the two strategies. The Fatty acid biosynthesis and TCA cycle pathways show that acetyl-CoA is the common precursor for both fatty acid and carboxylic acid synthesis (**Figure 5**). For Qd11 and Nm, low phosphorus promoted the expression of malate synthase (*AceB*) and citrate synthase (*CS*) genes, which enhanced the synthesis of carboxylic acids. However, the expression of fatty acid synthesis-related enzymes was reduced in Nm, which led to the downregulation of colonization by AMF in Nm. In contrast to Nm, Zh13 had elevated expression of acetyl-CoA carboxylase (*accA*), [acyl-carrier-protein] S-malonyltransferase (*FabD*), and fatty acyl-ACP thioesterase B (*FATB*) genes, which promoted fatty acid synthesis and ultimately mycorrhizal fungi symbiosis; however, the expression of carboxylic acid synthesis-related enzymes was reduced. This suggests that Nm uses acetyl-CoA more for the synthesis of carboxylic acids under low phosphorus availability. Zh13, however, chose to assign more acetyl-CoA to the pathway of fatty acid synthesis. We speculate that the direction of acetyl-CoA metabolism is a watershed in determining the choice of “outsourcing” and “do-it-yourself” strategies in soybean (**Figure 6**). Notably, TCA cycle pathways are carried out in mitochondria, whereas fatty acid synthesis takes place in the cytoplasm and plastids. In our study, several differentially expressed ALMT, MATE (**Table S5**), and ABC transporter proteins were identified. Although the subcellular localization of these organic transporter proteins is not yet clear, and the direction of transport of related metabolites cannot be determined, further studies will help to understand the mechanisms of strategic differentiation. In general, trade-offs occur among carboxylate exudation and mycorrhizal fungi symbiosis, but distinct species may have different trade-off mechanisms and equilibrium conditions.

Transcriptome analysis revealed that the number of DEGs decreased from 4049 in Qd11 to 1434 in Zh13, whereas only 701 DEGs were detected in Nm (**Figure 3C**), which is consistent with the trend in their phosphorus acquisition efficiency. Transcription factor analysis also showed a similar pattern. The most TFs were detected in the DEGs of Qd11 (361), followed by Zh13 (261), and the least TFs were detected in DEGs of Nm, with only 19 (**Table S4**). Similar results were observed in the comparative transcriptome analysis of rice (Prodhan et al., 2022) and *sorghum* (Zhang et al., 2019). This suggests that phosphorus-efficient varieties express more enzymes in response to low phosphorus. Under low phosphorus availability, Qd11 and Zh13 showed increased expression of the *PHT1*, *PAP*, and *PHR* genes (**Table S5**) and thus promoted phosphorus acquisition (Ham et al., 2018; Dissanayaka et al., 2021). Moreover, the expression of both fatty acid and carboxylic acid synthesis-related genes in Qd11 was markedly elevated. Thus, Qd11 was also the variety with the highest colonization by AMF, carboxylic acid exudation, and SRL, and eventually the highest phosphorus accumulation among the three varieties. ITPK1, INPP5B/F, VTC4, and PLC detected in the magenta, brown, and blue module may be involved in the degradation of inositol pyrophosphate (InsP) under low phosphorus treatment and may mediate the regulation of downstream IPS genes, especially the genes involved in the SPX-PHR system.

A series of transcription factors have been found in magenta modules. The ethylene signaling pathway plays an influential role in soybean in response to low phosphorus stress tolerance (Zhang et al., 2020). Under low phosphorus conditions, the transcription factors ERF1 can interact with the GCC box of target genes and regulate root growth, adventitious root, and root hair development (Iqbal et al., 2013; Pandey et al., 2021). EIN4 and ERF, both of which are implicated in ethylene signaling, were identified in the magenta module, and ERF demonstrated a high correlation with both SRL (*r* = 0.752) and RDT (*r* = -0.633). ERF1 and EIN4 may be involved in the regulation of root architecture in soybean in response to low phosphorus. Additionally, WRKY1 was found in the magenta module. In plants, WRKY transcription factors contribute to tolerance to abiotic stresses (Viana et al., 2018). WRKY transcription factors, in addition to regulating the transcription of PHT1;1 and PHO (Zhang et al., 2021), have also been demonstrated to influence root architecture (Li et al., 2021; Liu et al., 2022) by controlling the extracellular release of small molecules organic acids (Li et al., 2018; Shen et al., 2021) as well as the synthesis of jasmonic acid (Wang et al., 2018), which may play an instrumental role in controlling root morphology. Notably, the major members of the Magenta module are mainly involved in signal transduction, transcriptional regulation, and immune response; however, no carbohydrate metabolism-related processes were detected. Therefore, we speculate that the alteration of soybean root morphology under low phosphorus may mainly involve changes in cell shape but not cell division or proliferation, and thus the consumption of photosynthetic products may be low.

Compared to NM, the phosphorus-efficient variety synthesized a series of antioxidants [including proline (**Figure S4**), glutathione (**Figure S5**), carotenoid (**Figure S6**), flavonoid, and isoflavonoid (**Figures S7** and **S8**)], *via* metabolism to scavenge free radicals produced by the damaged membrane system caused by low phosphorus and maintain metabolic stability (Mo et al., 2019). In addition, these phosphorus-efficient varieties synthesized sulfoquinovosyl diacylglycerol and monogalactosyl-diacylglycerol instead of inadequate phospholipids, thereby improving phosphorus turnover and reuse (**Figure S9**; Yu et al., 2002; Rocha et al., 2018). We suggest that phosphorus-efficient varieties have accumulated more complex mechanisms of low phosphorus tolerance over a long evolutionary period relative to phosphorus-inefficient varieties such as Nm, which facilitates their better adaptation to low phosphorus environments (**Figure S10**).

## Conclusions

A wide variety of phosphorus acquisition strategies were observed in soybean varieties based on the analysis of 10 varieties. The root diameter and acetyl-CoA metabolic direction were found to be important factors in determining the phosphorus acquisition strategy of soybeans. Soybean varieties with thicker roots improved their phosphorus status under low phosphorus availability primarily by increasing their mycorrhizal symbiosis. Conversely, varieties with finer roots enhanced phosphorus acquisition by increasing their SRL and secreting more carboxylic acids. As indicated by transcriptomic analysis, acetyl-CoA metabolism determined whether photosynthetic assimilation products were converted into carboxylic acids or fatty acids, and this affected soybean mycorrhizal infestation and carboxylic acid emissions. Plant hormone signal transduction, plant-pathogen interaction, the MAPK signaling pathway, the glycolysis/gluconeogenesis pathway, and a series of important transcription factors (PHR, ERF1, and WRKY1) were involved in the regulation of phosphorus acquisition strategies. A natural progression of this work is to validate the role of acetyl-CoA metabolism in phosphorus acquisition strategies across species and explore the regulatory mechanisms of acetyl-CoA metabolism at the level of transcriptional and post-translational regulation. Our current findings do already enhance our understanding of phosphorus acquisition strategies in soybeans. In addition, they facilitate the development of more flexible breeding strategies to accommodate a wider variety of production scenarios in agriculture under low phosphorus environments.

## Data availability statement

The datasets presented in this study can be found in online repositories. The names of the repository/repositories and accession number(s) can be found in the article/**Supplementary Material**.

## Author contributions

SY, ZC, and JH designed the experiment. TY and SY performed the experiment. ZC and TY analyzed the data. ZC, TY, HD, RB, and JH wrote the manuscript. All authors contributed to the article and approved the submitted version.
