# Supplementary material for: Global transcriptomic analysis reveals candidate genes associated with different phosphorus acquisition strategies among soybean varieties
Source: Front Plant Sci. 2022 Dec 19;13:1080014. doi: 10.3389/fpls.2022.1080014 (PMC9806128; doi:10.3389/fpls.2022.1080014)
Supplement: Supplementary file 1 [file DataSheet_1.docx]

Fig. S1 Overview of the analysis pipelines inplemented in this study.


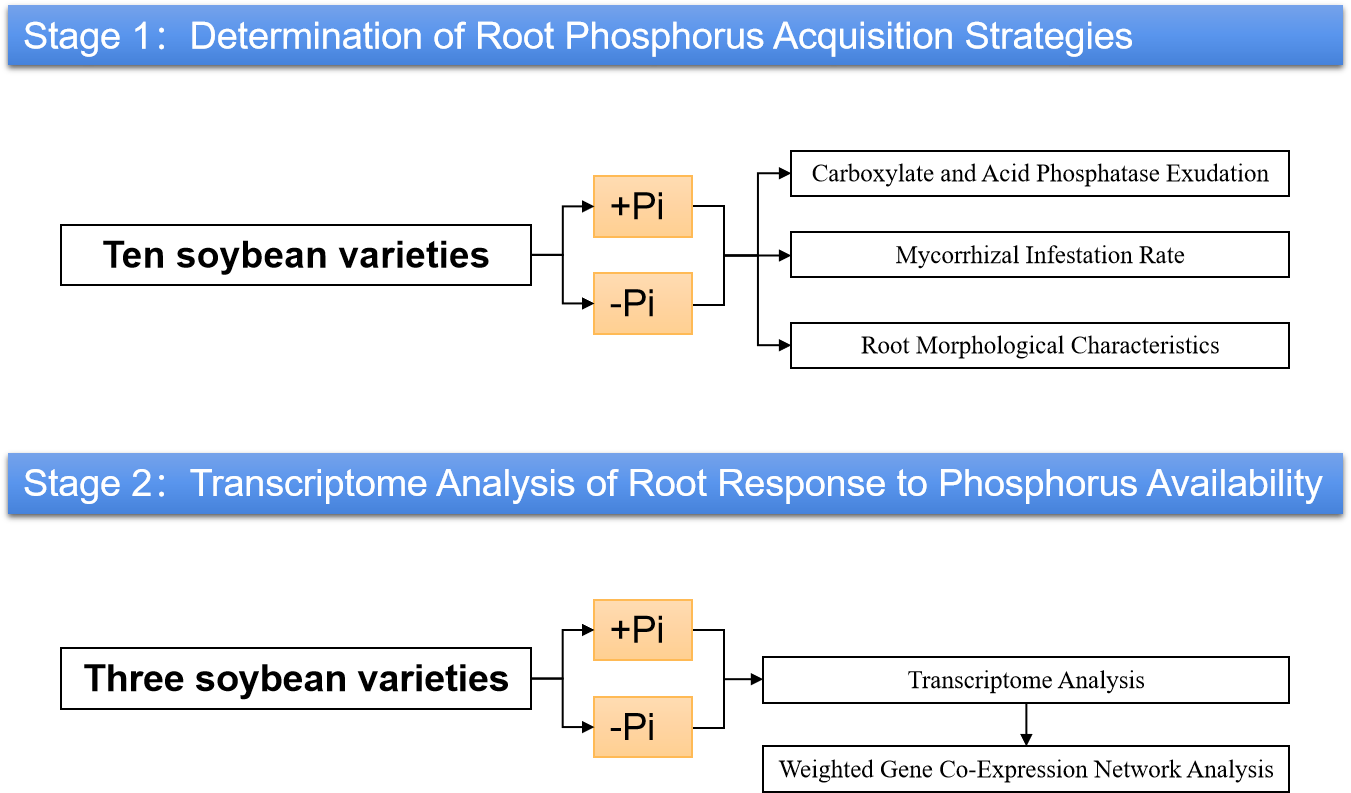


Fig. S2 Volcano plot for differentially expressed genes between (A) QD11_LP and QD11_NP; (B) ZH13_LP and ZH13_NP; (C) NM_LP and NM_NP. Variety abbreviation: Qd, Qian Dou 11; Zh13, Zhonghuang 13; Nm, Niu Mao Soybean; L, low soil phosphorus availability; N, normal soil phosphorus availability.


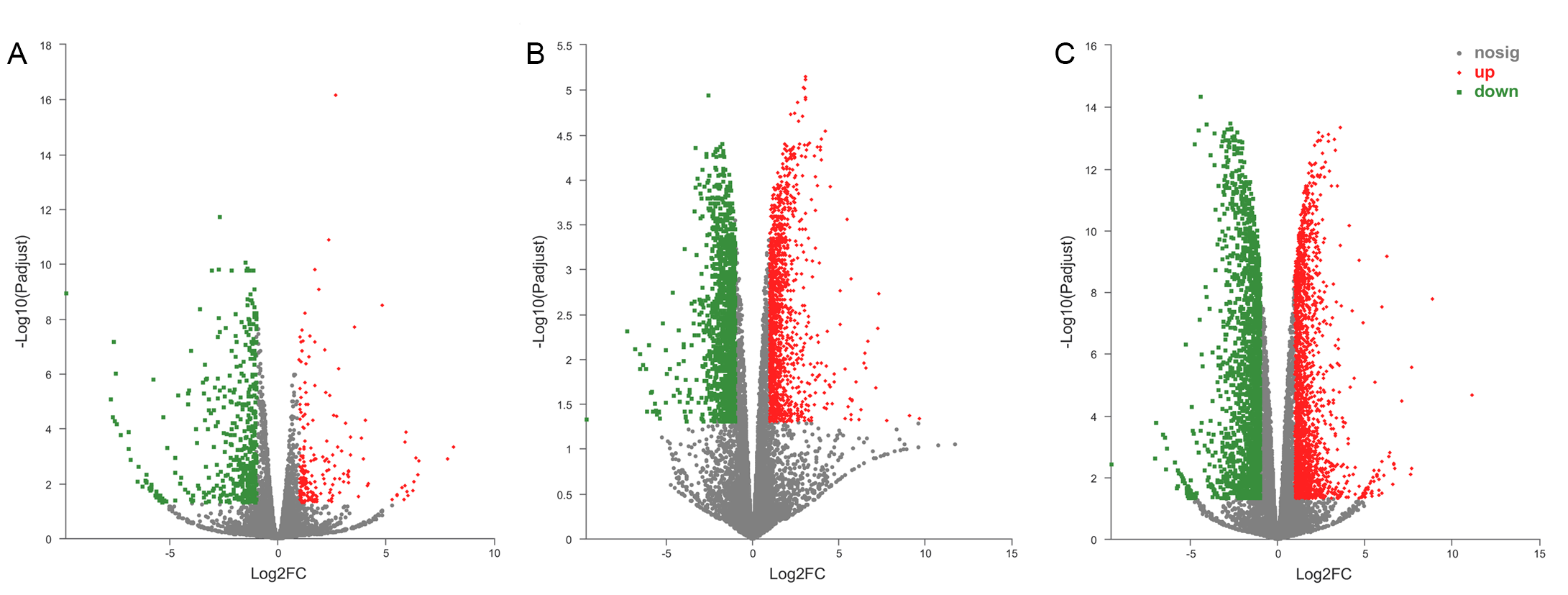


Fig. S3 KEGG enrichment of metabolites with differential gene sets. Species abbreviation: Qd, Qian Dou 11; Zh13, Zhonghuang 13; Nm, Niu Mao Soybean.


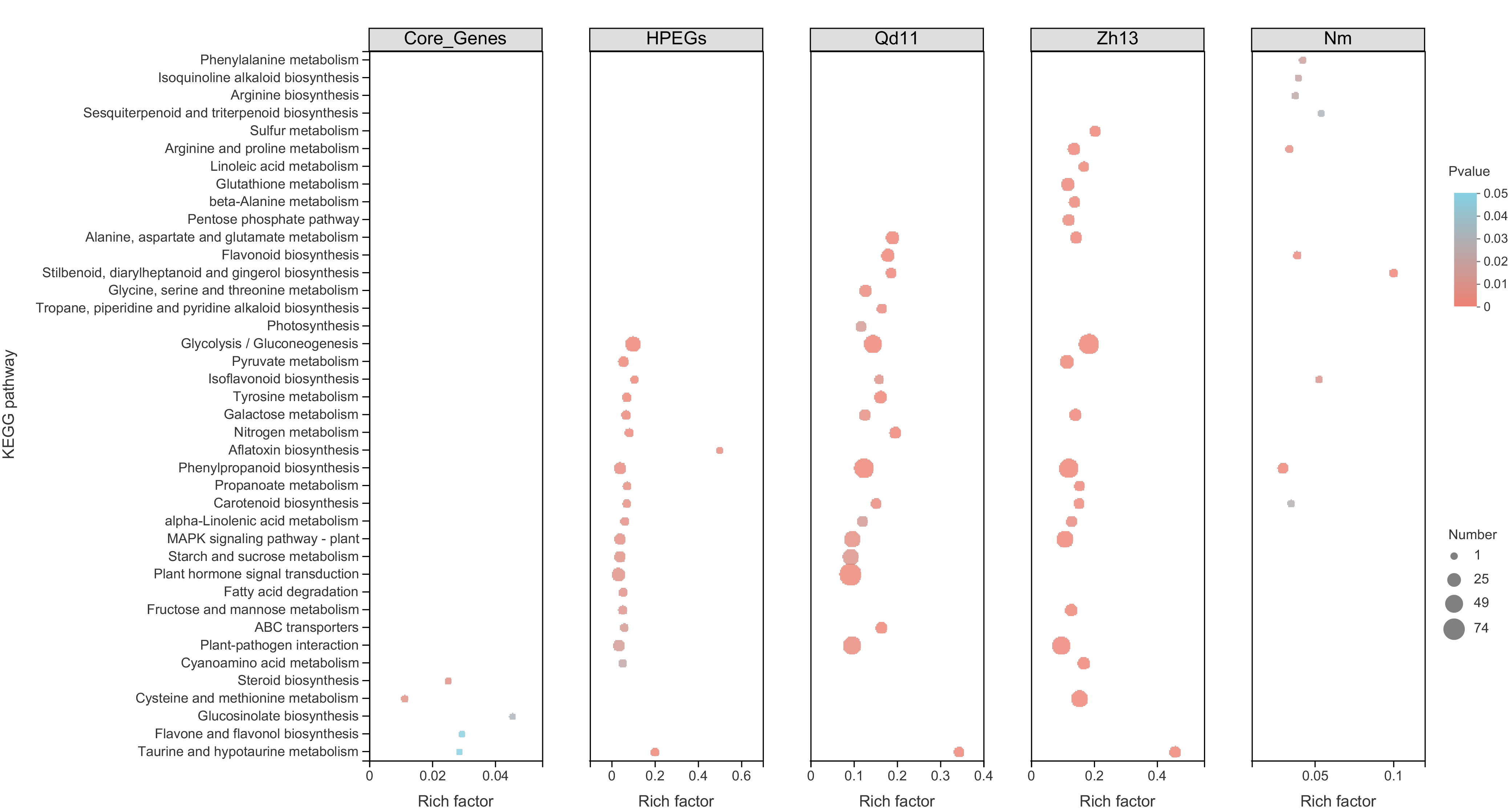


Fig. S4 Deferentially expressed genes associated with proline metabolism pathways. GLUD1_2, glutamate dehydrogenase; glnA, glutamine synthetase; P4HA, prolyl 4-hydroxylase. The heat map shows the expression patterns of deferentially expressed genes related to proline biosynthesis pathways in Qd11, Zh13, and NM at different P treatments. Enzyme names are shown in the gray circle. The color bar from blue to red is up-regulated; from red to blue is down-regulated. Variety abbreviation: Qd, Qian Dou 11; Zh13, Zhonghuang 13; Nm, Niu Mao Soybean; L, low soil phosphorus availability; N, normal soil phosphorus availability.


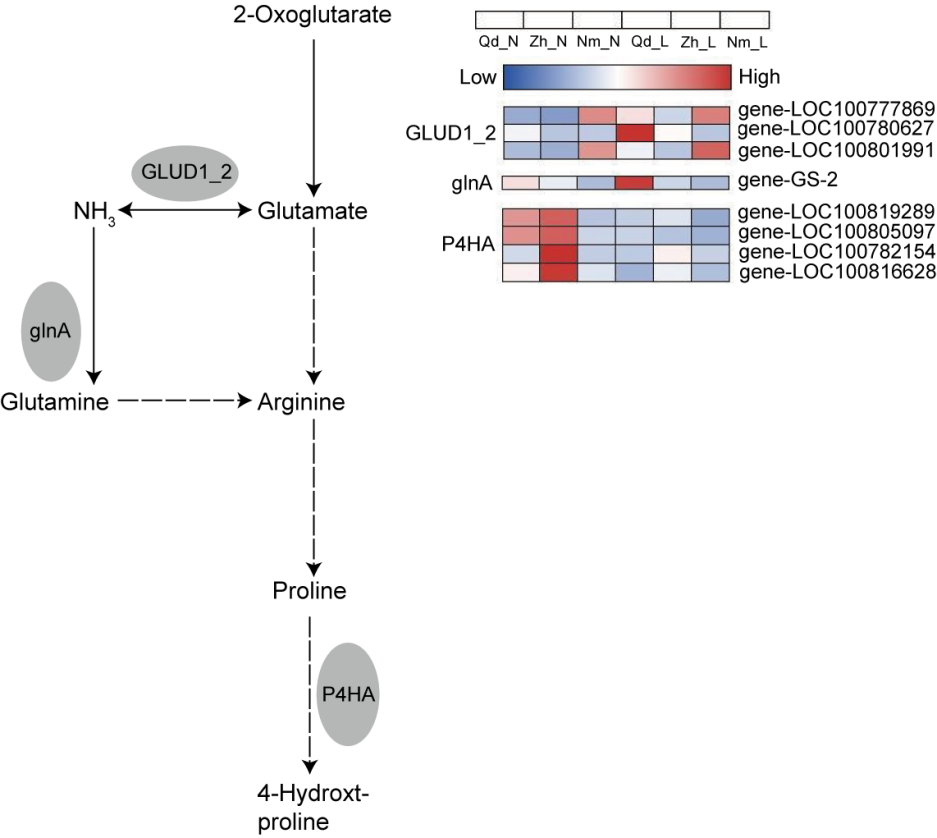


Fig. S5 Deferentially expressed genes associated with glutathione metabolism pathways. cysK, cysteine synthase; gpx, glutathione peroxidase; GGT1_5, glutathione hydrolase. The heat map shows the expression patterns of deferentially expressed genes related to glutathione metabolism pathways in Qd11, Zh13, and NM at different P treatments. Enzyme names are shown in the gray circle. The color bar from blue to red is up-regulated; from red to blue is down-regulated. Variety abbreviation: Qd, Qian Dou 11; Zh13, Zhonghuang 13; Nm, Niu Mao Soybean; L, low soil phosphorus availability; N, normal soil phosphorus availability.


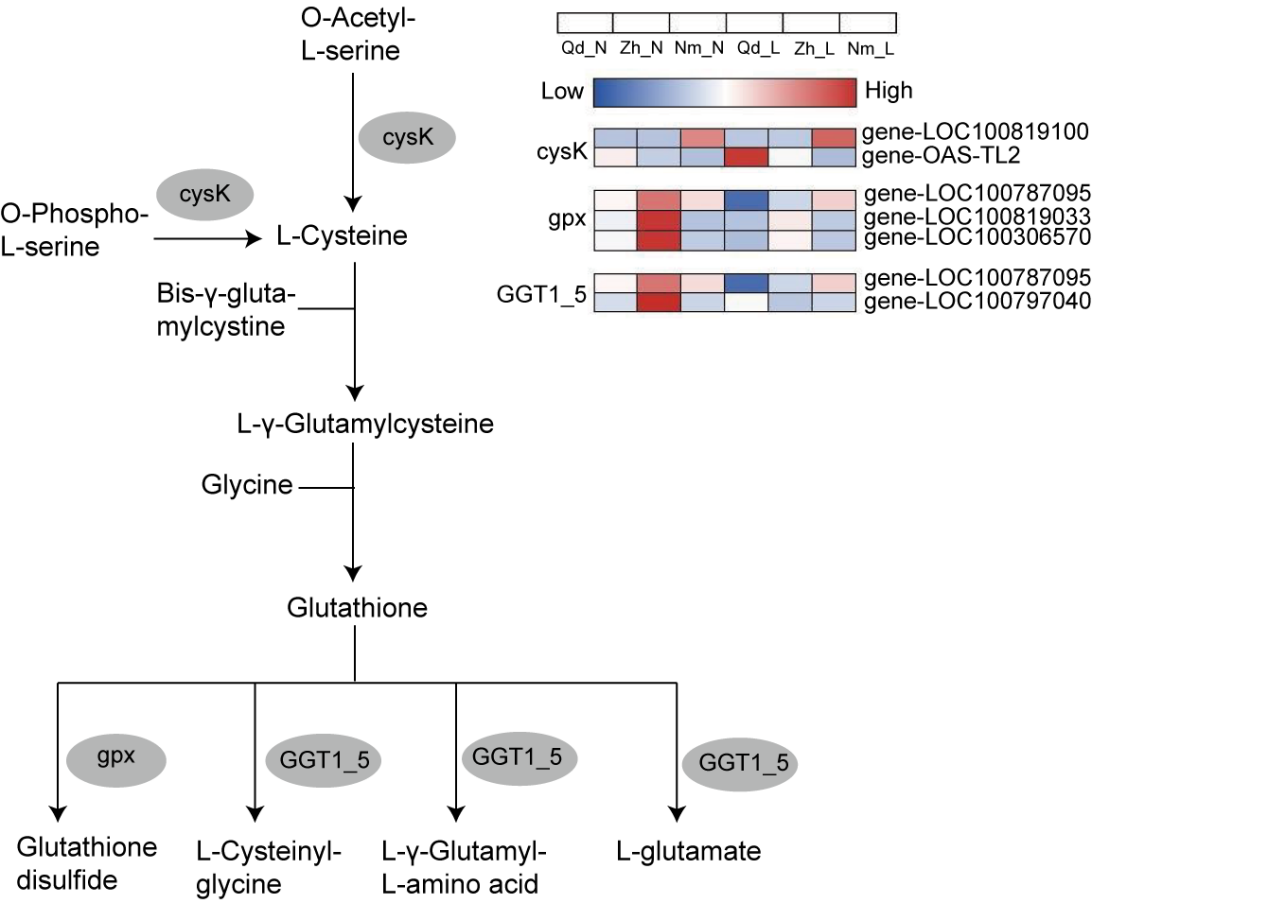


Fig. S6 Deferentially expressed genes associated with carotenoid biosynthesis pathways. crtB, 15-cis-phytoene synthase; Z-ISO, zeta-carotene isomerase; ZDS, zeta-carotene desaturase; crtZ, beta-carotene 3-hydroxylase; LUT5, beta-ring hydroxylase. The heat map shows the expression patterns of deferentially expressed genes related to Fatty acid biosynthesis and citrate cycle pathways in Qd11, Zh13, and NM at different P treatments. Enzyme names are shown in the gray circle. The color bar from blue to red is up-regulated; from red to blue is down-regulated. Variety abbreviation: Qd, Qian Dou 11; Zh13, Zhonghuang 13; Nm, Niu Mao Soybean; L, low soil phosphorus availability; N, normal soil phosphorus availability.


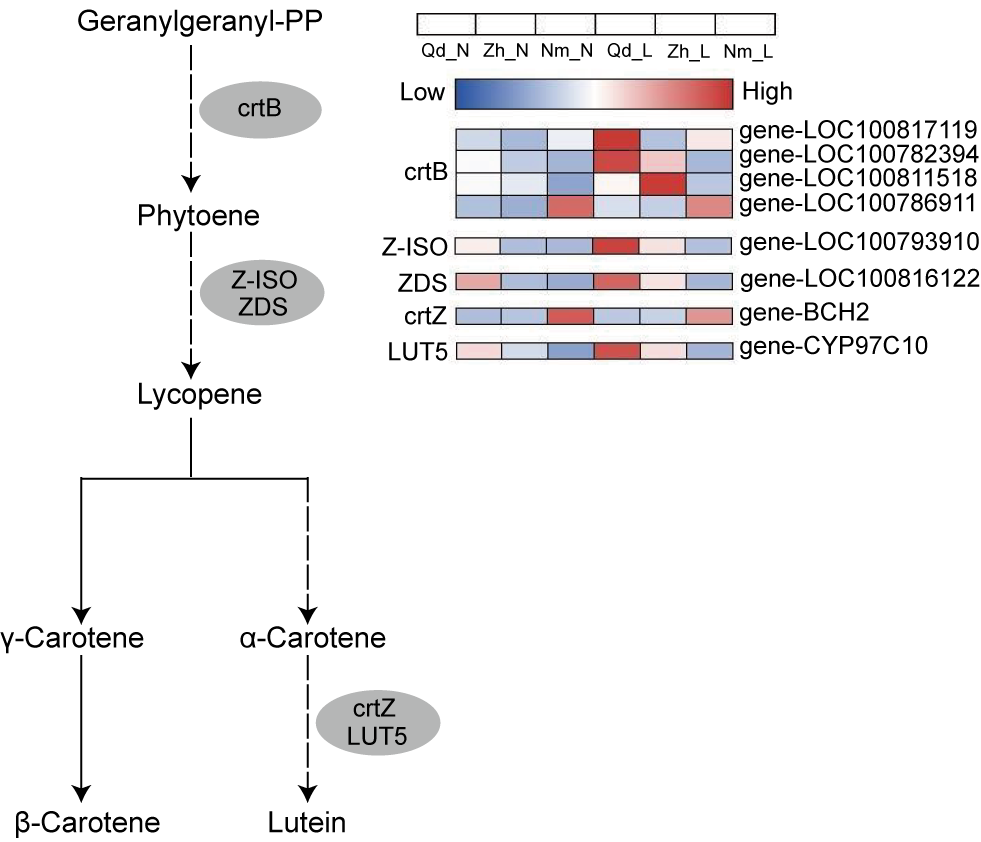


Fig. S7 Deferentially expressed genes associated with flavonoid biosynthesis pathways. CYP81E9, isoflavone 3'-hydroxylase; CYP93C, 2-hydroxyisoflavanone synthase; 7-IOMT, isoflavone-7-O-methyltransferase; CYP81E1_7, isoflavone / 4'-methoxyisoflavone 2'-hydroxylase. The heat map shows the expression patterns of deferentially expressed genes related to Fatty acid biosynthesis and citrate cycle pathways in Qd11, Zh13, and NM at different P treatments. Enzyme names are shown in the gray circle. The color bar from blue to red is up-regulated; from red to blue is down-regulated. Variety abbreviation: Qd, Qian Dou 11; Zh13, Zhonghuang 13; Nm, Niu Mao Soybean; L, low soil phosphorus availability; N, normal soil phosphorus availability.


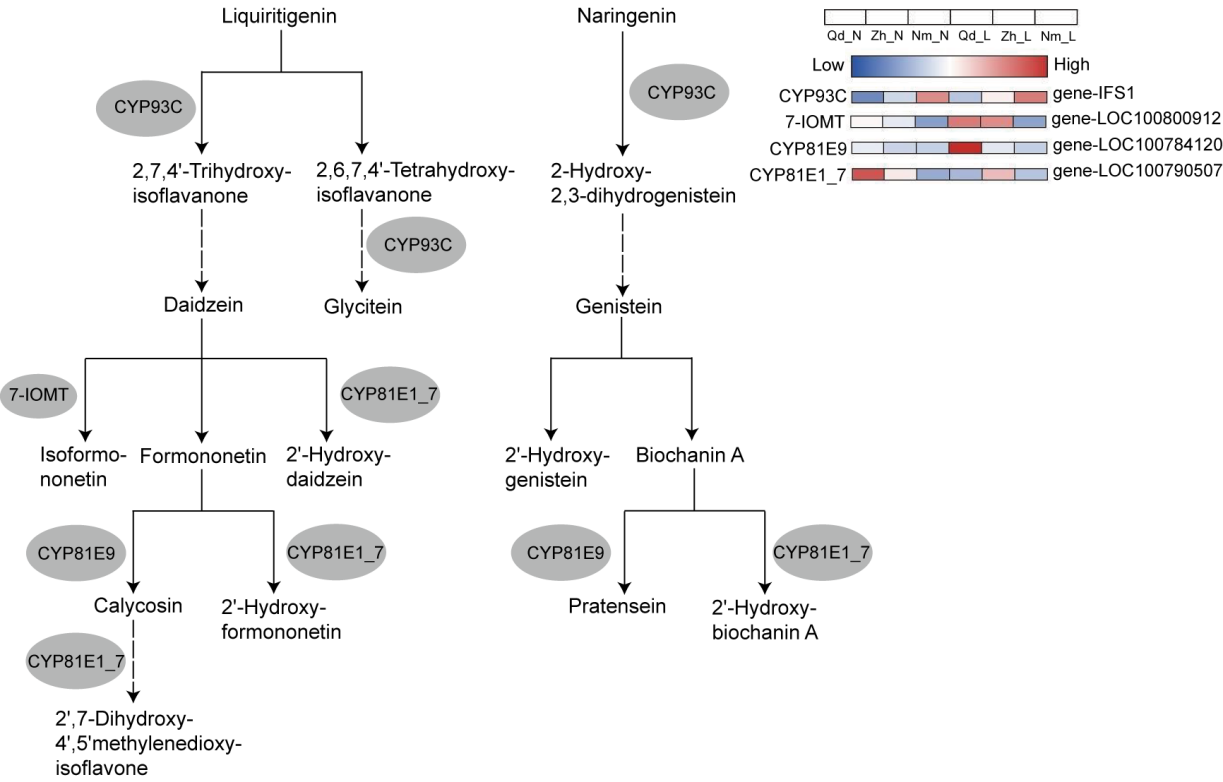


Fig. S8 Deferentially expressed genes associated with isoflavonoid biosynthesis pathways. CHS, chalcone synthase; CYP73A, trans-cinnamate 4-monooxygenase; E5.5.1.6, chalcone isomerase; E2.3.1.133, shikimate O-hydroxycinnamoyltransferase; CYP98A, 5-O-(4-coumaroyl)-D-quinate 3'-monooxygenase; PGT1, phlorizin synthase; CYP75B1, flavonoid 3'-monooxygenase; E2.1.1.104, caffeoyl-CoA O-methyltransferase; PKR, chalcone reductase. The heat map shows the expression patterns of deferentially expressed genes related to Fatty acid biosynthesis and citrate cycle pathways in Qd11, Zh13, and NM at different P treatments. Enzyme names are shown in the gray circle. The color bar from blue to red is up-regulated; from red to blue is down-regulated. Variety abbreviation: Qd, Qian Dou 11; Zh13, Zhonghuang 13; Nm, Niu Mao Soybean; L, low soil phosphorus availability; N, normal soil phosphorus availability.


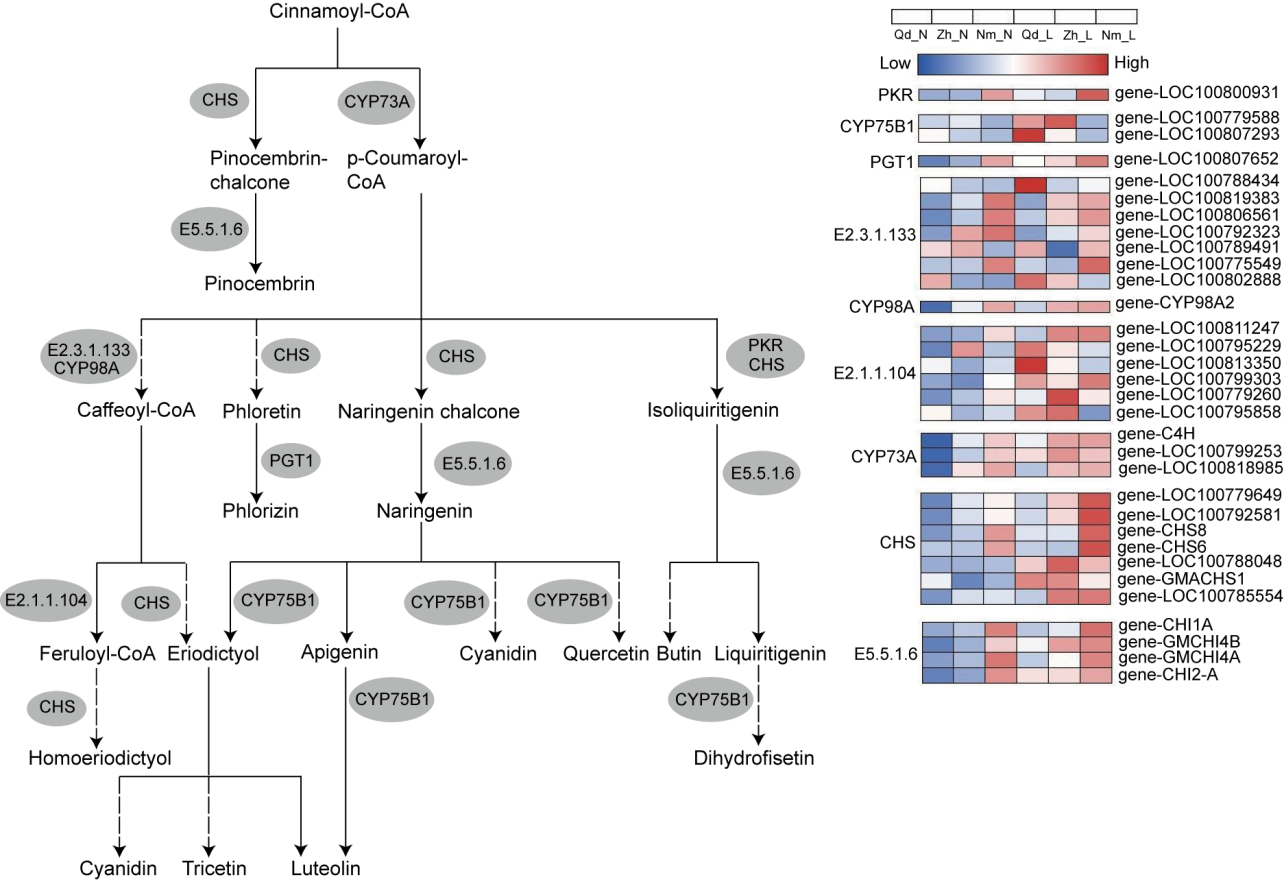


Fig. S9 Deferentially expressed genes associated with glycerolipid metabolism pathways. GPAT, glycerol-3-phosphate acyltransferase; ATS1, glycerol-3-phosphate O-acyltransferase; TGL4, TAG lipase / steryl ester hydrolase / phospholipase A2 / LPA acyltransferase; SQD2, sulfoquinovosyltransferase; E2.3.1.158, phospholipid:diacylglycerol acyltransferase; MGLL, acylglycerol lipase. The heat map shows the expression patterns of deferentially expressed genes related to Fatty acid biosynthesis and citrate cycle pathways in Qd11, Zh13, and NM at different P treatments. Enzyme names are shown in the gray circle. The color bar from blue to red is up-regulated; from red to blue is down-regulated. Variety abbreviation: Qd, Qian Dou 11; Zh13, Zhonghuang 13; Nm, Niu Mao Soybean; L, low soil phosphorus availability; N, normal soil phosphorus availability.


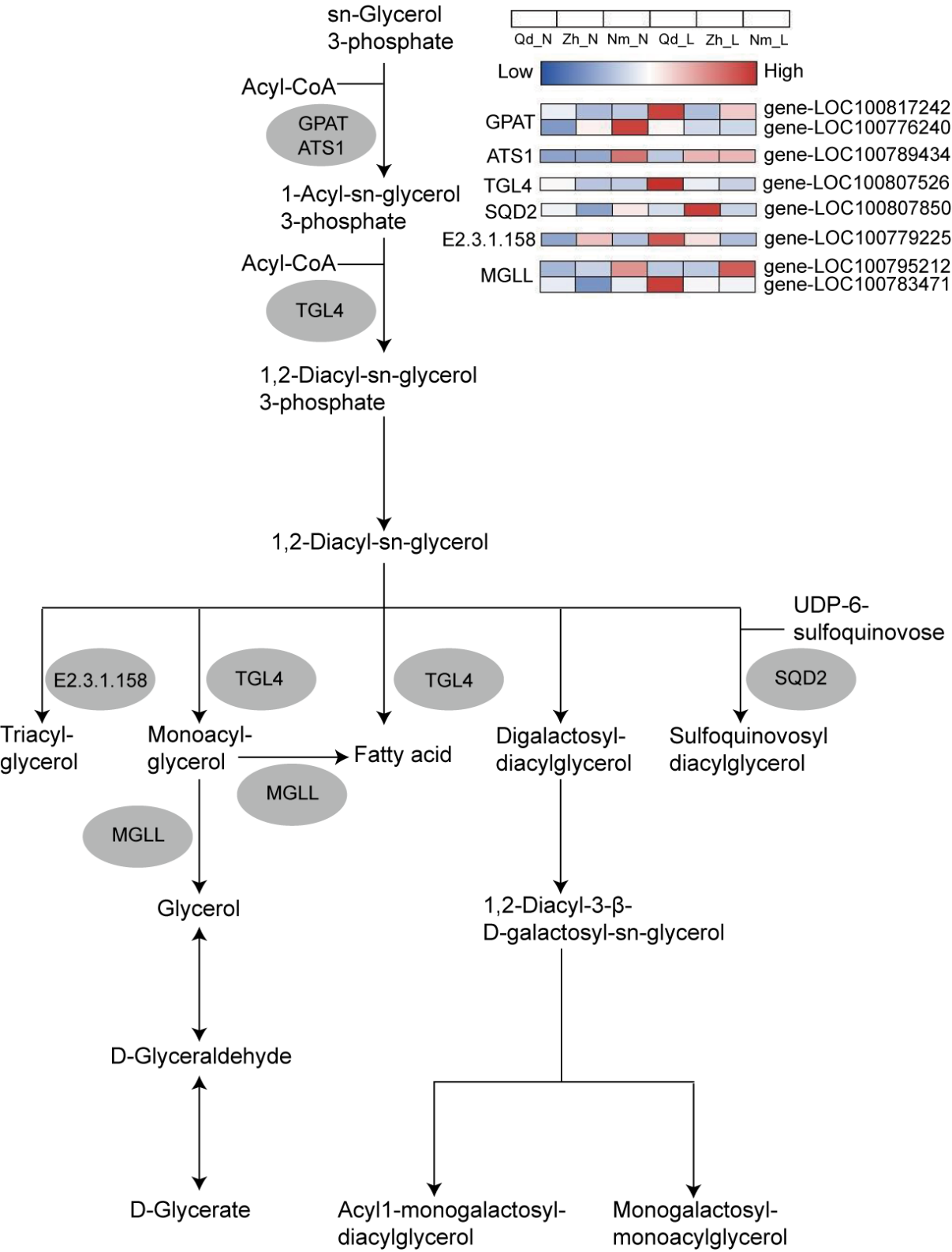


Fig. S10 KEGG enrichment of metabolites with differential varieties.


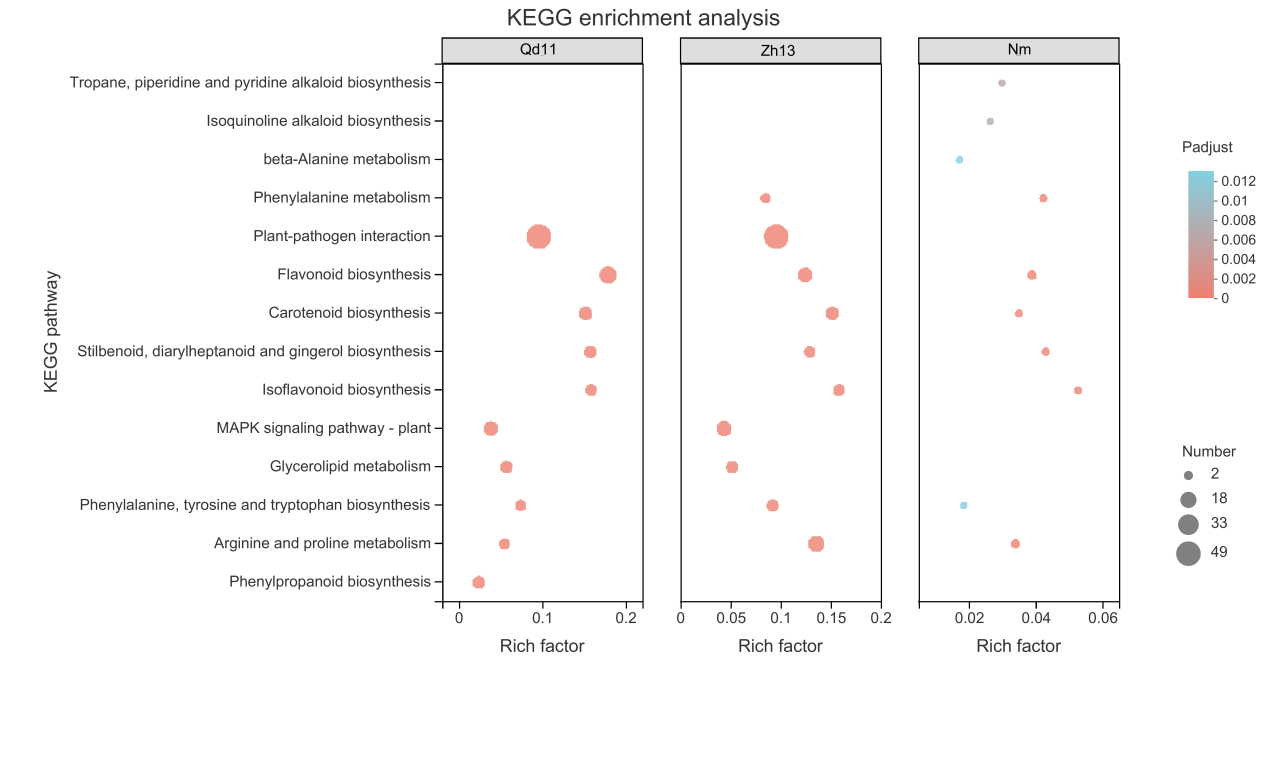


Table S1 Characteristic of tested soybean varieties.

| Varietie | Characteristic | Hundred-grain weight |
| --- | --- | --- |
| Qd11 | Phosphorus-efficiency variety;  High-yield variety in southwest China | 18-22 g |
| Sd | High-yield variety of China | 19.2 g |
| Nm | Phosphorus-inefficiency variety | 12-14 g |
| Zh13 | High-yield variety of China | 24-26 g |
| Hc6 | Phosphorus-efficiency variety | 19.9 g |
| Zh30 | Phosphorus-efficiency variety | 25.3 g |
| Hx3 | Phosphorus-efficiency variety | 17.5g |
| Hx2 | Phosphorus-efficiency variety | 18.8 g |
| Sy | High-yield variety in southwest China | 17 g |
| Zh301 | High-yield variety of China | 18.6-19.5 g |

Table S2 Effects of low phosphorus stress on root morphology of the 10 varieties. Each value is the mean (±SE) of three replicates.

| Varieties | root tissue density  (g cm^-3^) | | Root surface area  (cm^2^) | | root tips number | |
| --- | --- | --- | --- | --- | --- | --- |
|  | NP | LP | NP | LP | NP | LP |
| Qd11 | 0.16±0.01 | 0.12±0.01 | 153.96±0.57 | 162.12±1.25 | 815±27 | 521±19 |
| Zh30 | 0.15±0.01 | 0.15±0.01 | 178.01±1.25 | 179.4±6.55 | 1393±23 | 1683±11 |
| Zh301 | 0.20±0.01 | 0.14±0.01 | 191.33±3.25 | 240.06±8.54 | 836±33 | 846±30 |
| Zh13 | 0.15±0.01 | 0.16±0.01 | 217.04±3.56 | 240.63±6.52 | 662±22 | 1022±28 |
| Sd | 0.38±0.01 | 0.14±0.01 | 159.47±7.06 | 261.65±4.39 | 907±10 | 960±17 |
| Sy | 0.31±0.04 | 0.18±0.00 | 82.30±4.59 | 143.77±5.50 | 35197±30 | 3514±74 |
| Hx3 | 0.30±0.01 | 0.13±0.00 | 187.63±7.25 | 256.55±5.10 | 779±35 | 1219±40 |
| Hx2 | 0.31±0.02 | 0.19±0.01 | 180.66±4.14 | 151.86±8.15 | 776±30 | 3067±120 |
| Hc6 | 0.26±0.01 | 0.15±0.00 | 160.37±6.77 | 168.41±5.01 | 988±21 | 1037±34 |
| Nm | 0.12±0.00 | 0.14±0.00 | 196.61±4.87 | 171.39±1.87 | 704±25 | 872±11 |
| CV (%) | 37.75% | 12.22% | 23.34% | 22.84% | 72.12% | 72.94% |

Table S3 Effects of low phosphorus stress on carboxylates and acid phosphatase activity (APase) in the rhizosphere of the 10 varieties. Each value is the mean (±SE) of three replicates.

| Varieties | Malate (mg g^-1^ RDW) | | Citrate (mg g^-1^ RDW) | | Oxalate (mg g^-1^ RDW) | | Lactate (mg g^-1^ RDW) | | APase (μmol g^-1^ min^-1^) | |
| --- | --- | --- | --- | --- | --- | --- | --- | --- | --- | --- |
|  | NP | LP | NP | LP | NP | LP | NP | LP | NP | LP |
| Qd11 | 0.19±0.03 | 0.34±0.02 | 1.31±0.13 | 2.56±0.16 | 0.05±0 | 0.09±0 | 3.94±0.43 | 5.48±0.38 | 1.35±0.09 | 1.35±0.05 |
| Zh30 | 0.21±0.01 | 0.3±0.03 | 0.93±0.06 | 1.31±0.16 | 0.24±0.03 | 4.21±0.7 | 2.07±0.23 | 7.19±0.59 | 1.55±0.10 | 2.56±0.21 |
| Zh301 | 0.08±0.01 | 0.13±0.01 | 1.46±0.02 | 1.22±0.1 | 1.23±0.03 | 0.45±0.06 | 4.03±0.25 | 3.34±0.5 | 1.14±0.07 | 2.14±0.08 |
| Zh13 | 0.22±0.02 | 0.23±0.02 | 0.6±0.05 | 1.11±0.05 | 1.28±0.11 | 1.69±0.07 | 5.64±0.69 | 3±0.33 | 1.09±0.08 | 0.90±0.05 |
| Sd | 0.12±0.01 | 0.28±0.01 | 2.02±0.08 | 2.14±0.08 | 0.19±0.02 | 0.18±0.01 | 5.85±0.3 | 7.39±0.48 | 1.28±0.10 | 1.23±0.06 |
| Sy | 0.21±0.03 | 0.31±0.03 | 0.29±0.03 | 0.36±0.04 | 0.07±0.01 | 0.08±0 | 3.66±0.31 | 4.43±0.22 | 0.96±0.09 | 1.03±0.05 |
| Hx3 | 0.18±0.01 | 0.19±0.02 | 0.51±0.01 | 0.5±0.01 | 0.05±0.01 | 0.08±0.01 | 4.39±0.41 | 5.14±0.46 | 0.84±0.02 | 1.01±0.06 |
| Hx2 | 0.18±0.01 | 0.26±0.02 | 0.22±0.02 | 0.23±0.03 | 0.11±0.01 | 0.17±0.01 | 4.07±0.32 | 4.6±0.18 | 0.99±0.06 | 1.72±0.06 |
| Hc6 | 0.17±0.01 | 0.27±0.02 | 0.91±0.09 | 0.71±0.07 | 0.07±0 | 0.18±0 | 5.88±0.29 | 4.42±0.34 | 0.79±0.03 | 0.91±0.04 |
| Nm | 0.2±0.02 | 0.13±0.01 | 0.26±0.02 | 0.22±0 | 0.07±0 | 0.06±0 | 0.86±0.05 | 5.22±0.68 | 1.77±0.04 | 2.96±0.07 |
| CV (%) | 24.75% | 29.35% | 70.07% | 77.59% | 145.37% | 183.84% | 40.04% | 28.50% | 26.77% | 46.74% |

Table S4 Summary of the sequencing data generated for RNA-seq and mapping of the soybean genome. Variety abbreviation: Qd, Qian Dou 11; Zh13, Zhonghuang 13; Nm, Niu Mao Soybean; L, low soil phosphorus availability; N, normal soil phosphorus availability.

| Sample | Raw reads | Clean reads | Mapped reads |
| --- | --- | --- | --- |
| Nm_NP_1 | 50261584 | 49843448 | 43970438 |
| Nm_NP_2 | 55529578 | 55032052 | 47511113 |
| Nm_NP_3 | 54690672 | 54250514 | 47829157 |
| Nm_LP_1 | 51180238 | 50781780 | 47076377 |
| Nm_LP_2 | 51643340 | 51209074 | 47679154 |
| Nm_LP_3 | 58549434 | 58091334 | 53766238 |
| Qd11_NP_1 | 56525010 | 56068890 | 47348340 |
| Qd11_NP_2 | 52752382 | 52322844 | 44053337 |
| Qd11_NP_3 | 54249704 | 53735938 | 45303177 |
| Qd11_LP_1 | 50252740 | 49808662 | 45184484 |
| Qd11_LP_2 | 53837706 | 53389916 | 48367470 |
| Qd11_LP_3 | 49423316 | 49016176 | 44823923 |
| Zh13_NP_1 | 56580046 | 55230898 | 49713816 |
| Zh13_NP_2 | 54595398 | 54108824 | 48215032 |
| Zh13_NP_3 | 51973548 | 51492726 | 46417757 |
| Zh13_LP_1 | 54763544 | 54293654 | 48967823 |
| Zh13_LP_2 | 53424370 | 52960218 | 48025270 |
| Zh13_LP_3 | 58289508 | 57768374 | 52342145 |

Table S5 The number of transcription factors differentially expressed in gene sets. Variety abbreviation: Qd, Qian Dou 11; Zh13, Zhonghuang 13; Nm, Niu Mao Soybean.

| TF family | ALL DEGs | HPEGs | Qd11 | Zh13 | Nm |
| --- | --- | --- | --- | --- | --- |
| AP2 | 7 | 3 | 6 | 4 |  |
| ARF | 5 | 2 | 3 | 4 |  |
| B3 | 8 | 4 | 6 | 6 |  |
| BES1 | 2 |  | 1 |  |  |
| C2H2 | 10 | 5 | 8 | 7 |  |
| C3H | 7 | 1 | 5 | 3 |  |
| CAMTA | 2 | 1 | 2 | 1 |  |
| CO-like | 3 | 1 | 1 | 3 |  |
| DBB | 3 | 1 | 3 | 1 |  |
| Dof | 20 | 3 | 10 | 13 |  |
| E2F/DP | 1 |  |  | 1 |  |
| EIL | 1 | 1 | 1 | 1 |  |
| ERF | 85 | 27 | 61 | 47 | 5 |
| GATA | 5 |  | 5 |  |  |
| GRAS | 29 | 9 | 24 | 13 |  |
| GRF | 1 |  | 1 |  |  |
| HB-other | 34 | 4 | 25 | 13 |  |
| HD-ZIP | 5 | 1 | 4 | 2 |  |
| HSF | 6 | 3 | 4 | 5 |  |
| LBD (AS2/LOB) | 8 | 3 | 5 | 6 |  |
| MIKC | 7 | 3 | 5 | 5 |  |
| MYB | 56 | 7 | 40 | 21 | 2 |
| MYB_related | 30 | 2 | 14 | 15 | 3 |
| M_type | 9 | 3 | 5 | 7 |  |
| NAC | 40 | 13 | 35 | 18 |  |
| NF-YA | 1 |  | 1 |  |  |
| Nin-like | 3 | 1 | 2 | 2 |  |
| RAV | 2 | 1 | 2 | 1 |  |
| SBP | 5 | 1 | 1 | 5 |  |
| SRS | 2 | 1 | 2 | 1 |  |
| TALE | 3 | 1 | 3 | 1 |  |
| TCP | 7 |  | 2 | 5 |  |
| WRKY | 37 | 8 | 29 | 16 | 2 |
| ZF-HD | 1 |  | 1 |  |  |
| bHLH | 50 | 16 | 37 | 25 | 4 |
| bZIP | 15 | 1 | 7 | 9 |  |
| Total TFs | 510 | 127 | 361 | 261 | 19 |

Table S6 Candidate genes related to different soybean varieties. Variety abbreviation: Qd, Qian Dou 11; Zh13, Zhonghuang 13; Nm, Niu Mao Soybean.

| Gene collection | Gene family | Gene_id | Gene name | Gene description | FC | Padjust | Regulate |
| --- | --- | --- | --- | --- | --- | --- | --- |
| Qd | PHT | gene-GMPHT1_13 | GMPHT1;13 | phosphate transporter 1 family protein | 2.68 | 7.11E-09 | up |
|  |  | gene-GMPHT1_1 | GMPHT1;1 | phosphate transporter 1 family protein | 2.07 | 9.08E-09 | up |
|  |  | gene-GMPHT1_12 | GMPHT1;12 | phosphate transporter 1 family protein | 2.51 | 3.67E-08 | up |
|  |  | gene-GMPHT1_14 | GMPHT1;14 | phosphate transporter 1 family protein, transcript variant X1 | 0.49 | 4.64E-09 | down |
|  | PAP | gene-PAP14 | PAP14 | purple acid phosphatase-like, transcript variant X1 | 2.21 | 2.09E-09 | up |
|  |  | gene-LOC100817498 | LOC100817498 | purple acid phosphatase 22 | 3.01 | 5.72E-07 | up |
|  |  | gene-LOC100784006 | LOC100784006 | purple acid phosphatase 15, transcript variant X1 | 4.20 | 1.73E-03 | up |
|  | ALMT | gene-GMALMT34 | GMALMT34 | aluminum-activated malate transporter family protein | 0.36 | 1.65E-04 | down |
|  |  | gene-GMALMT5 | GMALMT5 | aluminum-activated malate transporter family protein | 0.48 | 3.24E-04 | down |
|  |  | gene-GMALMT16 | GMALMT16 | aluminum-activated malate transporter family protein | 0.17 | 2.55E-03 | down |
|  |  | gene-GMALMT7 | GMALMT7 | aluminum-activated malate transporter family protein | 0.02 | 1.22E-02 | down |
|  | ABC transporter | gene-LOC100804809 | LOC100804809 | ABC transporter G family member 17 | 3.04 | 9.29E-10 | up |
|  |  | gene-LOC100807709 | LOC100807709 | ABC transporter B family member 15-like | 0.46 | 6.12E-09 | down |
|  |  | gene-LOC100817829 | LOC100817829 | ABC transporter B family member 1 | 3.19 | 4.50E-08 | up |
|  |  | gene-LOC100777713 | LOC100777713 | ABC transporter B family member 4 | 2.92 | 7.09E-07 | up |
|  |  | gene-LOC100792425 | LOC100792425 | ABC transporter G family member 11 | 2.99 | 7.55E-07 | up |
|  |  | gene-LOC100776279 | LOC100776279 | ABC transporter G family member 14 | 0.49 | 3.70E-06 | down |
|  |  | gene-LOC100802547 | LOC100802547 | ABC transporter B family member 11, transcript variant X1 | 6.25 | 4.36E-06 | up |
|  |  | gene-LOC100777836 | LOC100777836 | ABC transporter C family member 3 | 3.89 | 1.25E-05 | up |
|  |  | gene-LOC100816700 | LOC100816700 | ABC transporter B family member 19 | 2.39 | 3.14E-05 | up |
|  |  | gene-LOC100800293 | LOC100800293 | ABC transporter B family member 11 | 2.19 | 9.41E-05 | up |
|  |  | gene-LOC102661014 | LOC102661014 | ABC transporter D family member 2, chloroplastic | 2.16 | 1.04E-04 | up |
|  |  | gene-LOC100808752 | LOC100808752 | ABC transporter C family member 4 | 2.66 | 8.17E-04 | up |
|  |  | gene-LOC100790313 | LOC100790313 | ABC transporter G family member 18 | 2.15 | 9.18E-04 | up |
|  |  | gene-LOC100780618 | LOC100780618 | ABC transporter B family member 19 | 0.15 | 3.24E-03 | down |
|  |  | gene-LOC100813711 | LOC100813711 | ABC transporter C family member 8, transcript variant X1 | 2.77 | 5.98E-03 | up |
|  |  | gene-LOC100807692 | LOC100807692 | ABC transporter G family member 15, transcript variant X1 | 0.28 | 2.13E-02 | down |
|  |  | gene-LOC100818527 | LOC100818527 | ABC transporter B family member 11, transcript variant X1 | 5.49 | 2.23E-02 | up |
|  | MATE | gene-MATE | MATE | aluminum-activated citrate transporter | 0.31 | 1.30E-08 | down |
|  | SPX2 | gene-GMSPX2 | GMSPX2 | SPX family protein SPX2, transcript variant X1 | 2.70 | 1.95E-05 | up |
|  | PHR | gene-GMPHR1 | GMPHR1 | MYB-CC domain-containing transcription factor PHR1, transcript variant X1 | 2.07 | 3.90E-02 | up |
| Zh | PHT | gene-GMPHT1_13 | GMPHT1;13 | phosphate transporter 1 family protein | 5.65 | 9.04E-05 | up |
|  |  | gene-GMPHT1_12 | GMPHT1;12 | phosphate transporter 1 family protein | 3.91 | 1.61E-04 | up |
|  |  | gene-GMPHT1_6 | GMPHT1;6 | phosphate transporter 1 family protein, transcript variant X1 | 0.32 | 1.62E-03 | down |
|  |  | gene-GMPHT1_11 | GMPHT1;11 | phosphate transporter 1 family protein | 3.65 | 1.97E-03 | up |
|  |  | gene-GMPHT1_7 | GMPHT1;7 | phosphate transporter 1 family protein | 0.48 | 1.03E-02 | down |
|  | ALMT | gene-GMALMT5 | GMALMT5 | aluminum-activated malate transporter family protein | 0.35 | 4.79E-04 | down |
|  |  | gene-GMALMT13 | GMALMT13 | aluminum-activated malate transporter family protein | 0.39 | 1.43E-03 | down |
|  |  | gene-GMALMT25 | GMALMT25 | aluminum-activated malate transporter family protein | 0.01 | 6.30E-03 | down |
|  | ABC transporter | gene-LOC100792531 | LOC100792531 | ABC transporter B family member 9, transcript variant X1 | 4.74 | 2.71E-04 | up |
|  |  | gene-LOC100804809 | LOC100804809 | ABC transporter G family member 17 | 3.69 | 2.81E-04 | up |
|  |  | gene-LOC100792425 | LOC100792425 | ABC transporter G family member 11 | 14.93 | 5.95E-04 | up |
|  |  | gene-LOC100779348 | LOC100779348 | ABC transporter C family member 3-like | 2.34 | 6.47E-04 | up |
|  |  | gene-LOC100776279 | LOC100776279 | ABC transporter G family member 14 | 0.34 | 6.94E-04 | down |
|  |  | gene-LOC102661014 | LOC102661014 | ABC transporter D family member 2, chloroplastic | 3.40 | 8.08E-04 | up |
|  |  | gene-LOC100801386 | LOC100801386 | ABC transporter G family member 36, transcript variant X2 | 2.35 | 1.97E-03 | up |
|  |  | gene-LOC100783266 | LOC100783266 | protein ABC transporter 1, mitochondrial | 2.78 | 2.04E-03 | up |
|  |  | gene-LOC100817665 | LOC100817665 | ABC transporter C family member 10, transcript variant X1 | 0.47 | 2.68E-03 | down |
|  |  | gene-LOC100807709 | LOC100807709 | ABC transporter B family member 15-like | 0.48 | 4.36E-03 | down |
|  |  | gene-LOC100777909 | LOC100777909 | ABC transporter B family member 21 | 0.37 | 4.48E-03 | down |
|  |  | gene-LOC100792991 | LOC100792991 | ABC transporter G family member 21 | 0.41 | 1.02E-02 | down |
|  |  | gene-LOC100777836 | LOC100777836 | ABC transporter C family member 3 | 3.26 | 1.29E-02 | up |
|  |  | gene-LOC100798801 | LOC100798801 | ABC transporter G family member 22, transcript variant X1 | 30.30 | 1.46E-02 | up |
|  |  | gene-LOC100816700 | LOC100816700 | ABC transporter B family member 19 | 2.65 | 2.17E-02 | up |
|  |  | gene-LOC100814181 | LOC100814181 | ABC transporter C family member 13, transcript variant X1 | 2.14 | 2.42E-02 | up |
|  |  | gene-LOC100790313 | LOC100790313 | ABC transporter G family member 18 | 2.54 | 3.52E-02 | up |
|  | SPX2 | gene-GMSPX2 | GMSPX2 | SPX family protein SPX2, transcript variant X1 | 2.75 | 6.64E-03 | up |
|  | PHR | gene-GMPHR20 | GMPHR20 | MYB-CC domain-containing transcription factor PHR20 | 2.72 | 5.65E-04 | up |
|  |  | gene-GMPHR29 | GMPHR29 | MYB-CC domain-containing transcription factor PHR29, transcript variant X1 | 2.54 | 4.43E-03 | up |
|  | miR399 | gene-MIR399B | MIR399B | microRNA MIR399b | 0.19 | 8.65E-03 | down |
| Nm | PHT | gene-GMPHT1_12 | GMPHT1;12 | phosphate transporter 1 family protein | 6.90 | 4.75E-08 | up |
|  |  | gene-GMPHT1_13 | GMPHT1;13 | phosphate transporter 1 family protein | 9.52 | 1.06E-04 | up |
|  | ALMT | gene-GMALMT34 | GMALMT34 | aluminum-activated malate transporter family protein | 2.14 | 4.71E-02 | up |
|  | ABC transporter | gene-LOC100804809 | LOC100792531 | ABC transporter B family member 9, transcript variant X1 | 2.15 | 1.61E-08 | up |
|  |  | gene-LOC100807709 | LOC100790313 | ABC transporter G family member 18 | 2.62 | 5.86E-05 | up |
|  |  | gene-LOC100817829 | LOC100804809 | ABC transporter G family member 17 | 4.07 | 1.04E-02 | up |
|  |  | gene-LOC100777713 | LOC100792425 | ABC transporter G family member 11 | 2.02 | 4.56E-02 | up |
|  | PHR | gene-GMPHR4 | GMPHR4 | MYB-CC domain-containing transcription factor PHR4, transcript variant X1 | 0.40 | 3.61E-03 | down |
|  | miR399 | gene-MIR399B | MIR399B | microRNA MIR399b | 10.10 | 4.89E-02 | up |

Table S7 KEGG enrichment analysis of modules

| **Module** | **Pathway id** | **KEGG Pathway** | **Pathway Category** | **Genes in module** | **Padjust** |
| --- | --- | --- | --- | --- | --- |
| **blue** | map00430 | Taurine and hypotaurine metabolism | Metabolism of other amino acids | 9 | 3.13×10-6 |
|  | map00195 | Photosynthesis | Energy metabolism | 15 | 4.88×10-6 |
|  | map04075 | Plant hormone signal transduction | Signal transduction | 38 | 1.54×10-4 |
|  | map04626 | Plant-pathogen interaction | Environmental adaptation | 27 | 4.07×10-4 |
|  | map00250 | Alanine, aspartate and glutamate metabolism | Amino acid metabolism | 11 | 1.53×10-3 |
|  | map04016 | MAPK signaling pathway - plant | Signal transduction | 21 | 1.90×10-3 |
|  | map00270 | Cysteine and methionine metabolism | Amino acid metabolism | 16 | 2.79×10-3 |
|  | map00910 | Nitrogen metabolism | Energy metabolism | 7 | 2.74×10-2 |
|  | map00908 | Zeatin biosynthesis | Metabolism of terpenoids and polyketides | 6 | 3.30×10-2 |
|  | map00750 | Vitamin B6 metabolism | Metabolism of cofactors and vitamins | 4 | 3.50×10-2 |
|  | map00480 | Glutathione metabolism | Metabolism of other amino acids | 11 | 3.59×10-2 |
|  | map00460 | Cyanoamino acid metabolism | Metabolism of other amino acids | 8 | 3.59×10-2 |
| **brown** | map00010 | Glycolysis / Gluconeogenesis | Carbohydrate metabolism | 23 | 5.08×10-13 |
|  | map00051 | Fructose and mannose metabolism | Carbohydrate metabolism | 7 | 7.73×10-3 |
|  | map00230 | Purine metabolism | Nucleotide metabolism | 9 | 8.80×10-3 |
|  | map00350 | Tyrosine metabolism | Amino acid metabolism | 6 | 9.88×10-3 |
|  | map00071 | Fatty acid degradation | Lipid metabolism | 7 | 9.93×10-3 |
|  | map00430 | Taurine and hypotaurine metabolism | Metabolism of other amino acids | 3 | 3.23×10-2 |
|  | map00710 | Carbon fixation in photosynthetic organisms | Energy metabolism | 6 | 3.38×10-2 |
|  | map00590 | Arachidonic acid metabolism | Lipid metabolism | 3 | 3.63×10-2 |
|  | map00620 | Pyruvate metabolism | Carbohydrate metabolism | 7 | 3.77×10-2 |
|  | map00330 | Arginine and proline metabolism | Amino acid metabolism | 5 | 4.64×10-2 |
|  | map00030 | Pentose phosphate pathway | Carbohydrate metabolism | 5 | 4.74×10-2 |
|  | map00040 | Pentose and glucuronate interconversions | Carbohydrate metabolism | 7 | 4.82×10-2 |
| **yellow** | map00941 | Flavonoid biosynthesis | Biosynthesis of other secondary metabolites | 15 | 2.15×10-12 |
|  | map00940 | Phenylpropanoid biosynthesis | Biosynthesis of other secondary metabolites | 19 | 1.91×10-7 |
|  | map00670 | One carbon pool by folate | Metabolism of cofactors and vitamins | 5 | 9.89×10-5 |
|  | map00943 | Isoflavonoid biosynthesis | Biosynthesis of other secondary metabolites | 5 | 2.25×10-4 |
|  | map00400 | Phenylalanine, tyrosine and tryptophan biosynthesis | Amino acid metabolism | 6 | 6.78×10-4 |
|  | map00360 | Phenylalanine metabolism | Amino acid metabolism | 5 | 6.28×10-4 |
|  | map00945 | Stilbenoid, diarylheptanoid and gingerol biosynthesis | Biosynthesis of other secondary metabolites | 5 | 5.88×10-4 |
|  | map00254 | Aflatoxin biosynthesis | Biosynthesis of other secondary metabolites | 2 | 5.52×10-4 |
|  | map00960 | Tropane, piperidine and pyridine alkaloid biosynthesis | Biosynthesis of other secondary metabolites | 5 | 4.81×10-4 |
|  | map00410 | beta-Alanine metabolism | Metabolism of other amino acids | 6 | 9.82×10-4 |
|  | map00260 | Glycine, serine and threonine metabolism | Amino acid metabolism | 6 | 4.45×10-3 |
|  | map04016 | MAPK signaling pathway - plant | Signal transduction | 10 | 5.32×10-3 |
| **turquoise** | map00940 | Phenylpropanoid biosynthesis | Biosynthesis of other secondary metabolites | 27 | 3.27×10-4 |
|  | map00460 | Cyanoamino acid metabolism | Metabolism of other amino acids | 10 | 1.12×10-2 |
|  | map00945 | Stilbenoid, diarylheptanoid and gingerol biosynthesis | Biosynthesis of other secondary metabolites | 7 | 2.16×10-2 |
|  | map00250 | Alanine, aspartate and glutamate metabolism | Amino acid metabolism | 9 | 3.63×10-2 |
| **green** | map00430 | Taurine and hypotaurine metabolism | Metabolism of other amino acids | 8 | 1.13×10-8 |
|  | map00270 | Cysteine and methionine metabolism | Amino acid metabolism | 12 | 3.21×10-5 |
|  | map00480 | Glutathione metabolism | Metabolism of other amino acids | 9 | 6.16×10-4 |
|  | map00250 | Alanine, aspartate and glutamate metabolism | Amino acid metabolism | 7 | 8.10×10-4 |
|  | map00920 | Sulfur metabolism | Energy metabolism | 5 | 3.02×10-3 |
|  | map00460 | Cyanoamino acid metabolism | Metabolism of other amino acids | 6 | 3.15×10-3 |
|  | map00910 | Nitrogen metabolism | Energy metabolism | 5 | 4.56×10-3 |
|  | map00260 | Glycine, serine and threonine metabolism | Amino acid metabolism | 6 | 1.00×10-2 |
|  | map00945 | Stilbenoid, diarylheptanoid and gingerol biosynthesis | Biosynthesis of other secondary metabolites | 4 | 1.08×10-2 |
|  | map00630 | Glyoxylate and dicarboxylate metabolism | Carbohydrate metabolism | 6 | 1.16×10-2 |
|  | map00908 | Zeatin biosynthesis | Metabolism of terpenoids and polyketides | 4 | 1.26×10-2 |
| **red** | map00010 | Glycolysis / Gluconeogenesis | Carbohydrate metabolism | 13 | 1.86×10-8 |
|  | map00270 | Cysteine and methionine metabolism | Amino acid metabolism | 8 | 2.06×10-4 |
|  | map00620 | Pyruvate metabolism | Carbohydrate metabolism | 7 | 6.11×10-4 |
|  | map00430 | Taurine and hypotaurine metabolism | Metabolism of other amino acids | 3 | 4.27×10-3 |
|  | map00640 | Propanoate metabolism | Carbohydrate metabolism | 3 | 4.51×10-2 |
